# Skeletal Muscle–Derived Extracellular Vesicles During Eccentric and Concentric Exercise Promote Muscle Regeneration After Injury

**DOI:** 10.1002/jcsm.70374

**Published:** 2026-08-28

**Authors:** Yining Zhou, Xiaoyan Shao, Pan Zhang, Jiaquan Lin, Xiang Chen, Xueying An, Zhuoying Jiang, Haosheng Wang, Depeng Fang, Yansi Xian, Bin Liu, Tao Shen, Ying Chen, Keran Li, Hongpeng Liu, Ying Li, Qing Jiang, Baosheng Guo

**Affiliations:** ^1^ Division of Sports Medicine and Adult Reconstructive Surgery, Department of Orthopedic Surgery, Nanjing Drum Tower Hospital, Affiliated Hospital of Medical School Nanjing University Nanjing Jiangsu China; ^2^ State Key Laboratory of Pharmaceutical Biotechnology Nanjing University Nanjing Jiangsu China; ^3^ Branch of National Clinical Research Center for Orthopedics, Sports Medicine and Rehabilitation Nanjing Jiangsu China; ^4^ Department of Rheumatology and Immunology Children's Hospital of Nanjing Medical University Nanjing China; ^5^ Department of Orthopedics, Air Force Hospital of Eastern Theater Command Nanjing University of Chinese Medicine Nanjing China; ^6^ Department of Orthopedics Nanjing Central Hospital Nanjing Jiangsu China

**Keywords:** eccentric exercise, extracellular vesicles, lipid metabolites, muscle regeneration

## Abstract

**Background:**

Skeletal muscle injuries significantly impair mobility and function, yet effective therapeutic interventions remain limited. Both eccentric exercise (EE) and concentric exercise (CE) promote muscle repair, but the underlying mechanisms are not fully understood. Muscle‐derived extracellular vesicles (mEVs) have emerged as critical mediators of intercellular communication; however, their role in exercise‐induced regeneration remains unclear.

**Methods:**

A murine model of barium chloride‐induced muscle injury was used to compare the effects of EE and CE on muscle regeneration. mEVs were isolated from sedentary (SED), CE‐ and EE‐conditioned muscle and characterised by nanoparticle tracking analysis, transmission electron microscopy and western blotting. Metabolic profiling of mEVs was performed using LC–MS. The functional roles were assessed through intramuscular injection of mEVs and GW4869‐mediated inhibition of mEV secretion. The effects of mEVs on myogenesis were further examined in C2C12 myoblasts.

**Results:**

EE significantly enhanced muscle regeneration compared with CE, as evidenced by improved histology, reduced fibrosis (*F* (2,15) = 59.37, *p* < 0.0001) and increased expression of myogenic markers such as Myod (*p* < 0.001), Myog (*p* < 0.001) and eMyhc (*p* < 0.001). EE also induced greater release of mEVs than CE, as indicated by higher expression of mEV markers and Rab27a/b (Rab27a, *p* < 0.001; Rab27b, *p* = 0.1222). Inhibition of mEV secretion with GW4869 abolished the regenerative benefits of exercise. Exogenous administration of EE‐mEVs enhanced muscle repair and C2C12 differentiation (Myod, *F* (2, 6) = 33.09, *p* < 0.001; Myog, *F* (2, 6) = 66.41, *p* < 0.001) more effectively than CE‐mEVs or SED‐mEVs. Metabolomic analysis revealed significant enrichment of lipid metabolites in EE‐mEVs (*N* = 5, *p* < 0.05), which was consistent with the upregulation of lipid metabolism–related genes. RNA‐seq analyses further indicated that lipid metabolites enriched in mEVs contributed to muscle repair potentially through activation of energy‐sensing pathways such as AMPK.

**Conclusions:**

EE facilitates muscle repair more effectively than CE by promoting the release of mEVs enriched in pro‐regenerative lipid metabolites. These findings suggest EE‐mEVs as a promising biological therapeutic strategy for muscle injury, particularly in cases where exercise is not feasible.

## 1 Introduction

Skeletal muscle injuries are prevalent in both athletic and general populations, often resulting from acute trauma or repetitive strain [1], and leading to severely restricted limb activity. Recent evidence‐based clinical studies [2] have shown that muscle contraction exercises, including eccentric exercise (EE) and concentric exercise (CE) [3], can effectively promote muscle repair [4]. EE generates higher mechanical tension with lower metabolic cost than CE. This unique stress profile potently activates regenerative signalling [5], suggesting that EE offers superior therapeutic advantages for muscle recovery. However, the underlying mechanisms through which these exercises promote muscle regeneration are still unclear.

Currently, numerous studies are focused on elucidating the molecular mechanisms by which exercise promotes muscle regeneration. In addition to directly promoting satellite cell (SC) self‐renewal [6] and differentiation [7], exercise induces skeletal muscle to release myokines that participate in muscle regeneration. For example, exercise releases Musclin, which reduces fibro‐adipogenic progenitors (FAPs) by upregulating FILIP1L, thereby promoting muscle regeneration [8], and also increases the myokine CLCF1 to enhance glucose uptake, glycolytic flux and mitochondrial metabolism [9]. Emerging evidence indicates that skeletal muscle actively releases extracellular vesicles (EVs, 50–150 nm) during both EE and CE. These EVs serve as critical mediators [10] of inter‐tissue communication, transporting bioactive molecules to orchestrate systemic adaptations to physical activity [11]. However, whether skeletal muscle–derived EVs (mEVs) released during CE participate in muscle repair remains to be elucidated.

Previous studies have reported that mEVs play an important role in muscle regeneration, with their contents shown to promote muscle repair processes. For example, myogenic EV miR‐140‐5p modulates skeletal muscle regeneration and injury repair by regulating muscle SCs [12]. Additionally, the levels of miR‐133a, miR‐133b, miR‐1 and miR‐206 in mEVs are significantly increased during the proliferation and differentiation of skeletal muscle cells [13]. Importantly, mEVs act as effective mediators of communication from skeletal muscle fibres to SCs, regulating their differentiation, proliferation and metabolism [14]. Our previous study showed that the mechanism underlying sarcopenia involves atrophic skeletal muscle fibre–derived small EVs containing miR‐690, which inhibits SC differentiation during ageing [15]. However, the functional role of secreted mEVs during exercise in regulating muscle regeneration remains to be explored.

In this study, we demonstrated that both EE and CE promote muscle repair after injury. Notably, EE induced a greater release of mEVs than CE. Metabolomic profiling further revealed distinct cargo compositions between EE‐mEVs and CE‐mEVs, with EE‐mEVs exhibiting significant enrichment in lipid metabolites. This finding was consistent with the concomitant upregulation of lipid metabolism‐related genes observed via quantitative PCR (qPCR). Based on these results, we propose that EE‐mEVs may represent a promising natural therapeutic strategy for enhancing muscle recovery in individuals unable to perform exercise post‐injury.

## 2 Materials and Methods

### 2.1 Animal Experiments

The male C57BL/6 mice (8 weeks old, 20–25 g) were acquired from Nanjing Ziyuan Biotechnology Co. Ltd. (Nanjing, China). For the Aurora Scientific 1200A system, mEVs secreted from SOL or EDL muscles (*n* = 10). For all remaining animal experiments, the number of animals used in each group is specified in the corresponding figure legends. All animal experiments were approved by the Institutional Animal Ethics Committee of Nanjing University and conducted in accordance with its Guidelines for the Care and Use of Laboratory Animals.

### 2.2 Muscle Injury

To induce acute muscle damage, 50 μL of 1.2% Barium Chloride was injected into the tibialis anterior (TA) muscle of a randomly selected limb in male C57BL/6 mice using a single multipoint injection [16]. The control strategies were separated based on the subsequent experimental models:

For the ex vivo muscle contraction model (Aurora Scientific system): The contralateral limb was injected with an equal volume of saline solution and served as the internal baseline control.

For the treadmill exercise model: Mice underwent unilateral injury only. To eliminate potential systemic confounding effects from exercise, the contralateral limb was not used as a control. Instead, a dedicated separate group of completely uninjured mice (with neither limb modelled) was established to serve as the true baseline control.

Throughout the procedure, mice were maintained under isoflurane anaesthesia. Preliminary experiments were conducted to confirm the feasibility of this approach.

### 2.3 Exercise Protocol

For treadmill exercise after acute muscle injury, mice were divided into three groups: the sedentary (SED) group, the CE group and the EE group. CE is uphill‐run training; EE is downhill‐run training [17]. Forty‐eight hours after acute injury, mice were first acclimated for 15 min at 5 m/min at a ± 15° incline, with EE mice exercising at −15° (downhill) and CE mice at +15° (uphill). They were then exercised for 20 min at 12 m/min at the same respective inclines for 2 days. After the period, the mice were directly exercised for 45 min at a speed of 12 m/min at a ± 15° incline for 3 continuous days [18].

For ex vivo muscle exercise, we use the ex vivo muscle contraction system to mimic CE and EE of soleus (SOL) or extensor digitorum longus (EDL). Under isoflurane anaesthesia, the SOL or EDL muscles were quickly removed from the mice, and then, we followed the Aurora Scientific 1200A muscle test system manual to soak the muscle in Krebs solution (G0430, Solarbio). Following this, we find the optimal muscle length and optimal stimulation intensity, and then start the contraction programs (listed in Table S1).

### 2.4 Tissue Collection and Processing

For tissue collection, mice were euthanised on Day 7 after BaCl_2_‐induced injury, following completion of the respective interventions. To accommodate distinct downstream applications, the harvested TA muscles were divided into two portions. One portion was immediately snap‐frozen in liquid nitrogen and stored at −80°C for subsequent molecular analyses. The remaining portion was fixed in 4% paraformaldehyde (PFA) to facilitate downstream tissue dehydration, embedding and histological sectioning.

### 2.5 mEVs Isolation

For mEVs isolated by enzymatic digestion, the male C57BL/6 mice (8 weeks old) were randomly divided into three groups, which were the SED, CE and EE groups. The CE and EE groups underwent continuous uphill or downhill running for 40 min at a speed of 12 m/min and a ± 15° incline for 6 weeks. Then, we isolated the fresh TA muscle from mice and rinsed them thoroughly with PBS and minced into 1–2 mm^3^ pieces. The tissue fragments were then digested with collagenase D (2 mg/mL) and DNase I (40 U/mL) at 37°C with gentle agitation (50 rpm) for 1 h, followed by PBS addition to terminate the digestion. The resulting filtrate was subsequently subjected to gradient centrifugation. The gradient centrifugation procedure [19] consisted of sequential centrifugation steps at 300×g for 10 min to remove large debris followed by 2000×g for 20 min to collect the supernatant. The supernatant was then centrifuged at 10 000×g for 30 min, after which the resulting supernatant was collected. Finally, the sample was ultracentrifuged at 100 000×g for 2 h. The final pellet was collected and resuspended in 50 μL PBS. EV protein concentration was quantified using a BCA Protein Assay Kit (P0011, Beyotime Biotech). For all in vivo functional experiments, mEV administration was normalised based on total EV protein content. An equal amount of mEV protein (25 μg per muscle) was administered across all groups (SED‐mEVs, CE‐mEVs and EE‐mEVs).

For mEVs isolated by the Aurora Scientific 1200A system, SOL or EDL muscles were prepared as described previously, immersed in Krebs solution and subjected to the corresponding contraction procedure following the Aurora Scientific 1200A muscle test system manual. Subsequently, the Krebs solution containing mEVs secreted from SOL or EDL muscles (*n* = 10) was collected, and subsequent ultracentrifugation steps were performed as described for the in vivo samples above.

All mEVs were isolated from muscles of independently assigned mice in each group, and contralateral saline‐injected limbs were not used for EV isolation.

### 2.6 Cell Culture

Mouse C2C12 myoblasts obtained from ATCC were cultured in DMEM supplemented with 10% foetal bovine serum (FBS, Gibco) and 1% penicillin–streptomycin at 37°C in 5% CO_2_. To investigate the effects of mEVs derived from different exercise modalities on myoblasts, the cells were treated with 20 μg/mL of mEVs for 48 h prior to subsequent experiments assessing the expression of relevant indicators.

### 2.7 Transmission electron Microscopy (TEM) Analysis

Carbon‐coated grids were glow‐discharged in air for 1 min prior to use. A 10‐μL sample was then applied to the grid, followed by immediate negative staining with 2% phosphotungstic acid for 60 s. Finally, the prepared grids were imaged under an H‐7800 transmission electron microscope at an accelerating voltage ranging from 80 to 120 kV.

### 2.8 Analysis of Nanoparticle Tracking Analyser (NTA)

mEVs were characterised using NTA (NanoSight NS300, Malvern Panalytical) to determine particle size distribution and concentration. Briefly, EV samples were diluted in PBS to achieve an optimal concentration range of 10^6^–10^9^ particles/mL, ensuring minimal particle overlap while maintaining detection sensitivity. Samples were loaded into a laser‐equipped chamber, and Brownian motion of individual particles was tracked via scattered light captured by a CCD camera at 25 frames per second. Particle trajectories were analysed using the NTA software [20].

### 2.9 In Vivo Treatment With mEVs and GW4869

GW4869 was dissolved in DMSO to a concentration of 10 μg/μL as a stock solution, and we used 0.9% normal saline to dissolve the stock solution to ensure that the total amount of GW4869 injected into TA muscle was 1 μg. The TA muscles of mice were intramuscularly injected with 20 μL of normal saline or GW4869 every other day for 14 days. The mEVs were dissolved in PBS and intramuscularly injected with 20 μL of PBS or mEVs every other day after muscle damage [15].

### 2.10 H&E Staining

Muscle tissues fixed in 4% PFA were dehydrated through a graded ethanol series, cleared in xylene and embedded in paraffin. Sections (5–10 μm) were prepared and dried. For staining, sections were dewaxed and rehydrated, followed by staining with haematoxylin and eosin Y. After dehydration and clearing, sections were mounted with neutral medium.

### 2.11 Immunofluorescence Staining

Immunofluorescence staining was performed as described [21], followed by overnight incubation with primary antibodies (listed in Table S2) and the appropriate secondary antibodies (Invitrogen, Guangzhou, China). Finally, the slides were mounted with DAPI‐containing anti‐fade mounting medium.

### 2.12 Immunohistochemical Staining

Dewaxed and rehydrated sections were immersed in EDTA antigen retrieval solution and heated for 10 min for antigen retrieval. The sections were then allowed to cool naturally to room temperature before being incubated in 3% hydrogen peroxide for 15 min. All sections were blocked with QuickBlock Immunostaining Blocking Buffer for 1 h and then incubated with the primary antibody at 4°C overnight. On the following day, the sections were incubated with the secondary antibody, followed by DAB staining.

### 2.13 Western Blots

Tissue proteins were extracted using RIPA lysis buffer and quantified with a BCA assay kit. Proteins were denatured in 5× SDS loading buffer at 95°C for 5 min, separated on 10% SDS‐PAGE gels and transferred to PVDF membranes. After blocking with rapid blocking buffer, membranes were incubated with primary antibodies overnight at 4°C with shaking. Following washes, membranes were incubated with corresponding secondary antibodies and signals were detected using an ECL substrate kit (Tanon, China).

### 2.14 RNA Extraction and RT‐qPCR

Total RNA was isolated using TRIzol reagent. cDNA was synthesised with HiScript IV RT SuperMix following the manufacturer's protocol. qPCR was performed on a QuantStudio 5 Real‐Time PCR System (Thermo Fisher Scientific) using ChamQ SYBR qPCR Master Mix in 384‐well plates. All reactions were carried out under standard cycling conditions. Relative gene expression was calculated using the 2−ΔΔCT method. Primer sequences are provided in Table S3.

### 2.15 Metabolomics Analysis

Metabolomic analysis was performed on mEVs isolated from the SED, CE and EE groups. Each biological sample consisted of mEVs pooled from five mice, with three technical replicates per group. Samples were centrifuged at 12 000 rpm (13 800×g) for 15 min at 4°C. The supernatant was collected, dried and reconstituted in methanol:acetonitrile: water (2:2:1, v/v/v) supplemented with isotope‐labelled internal standards. After vortexing and repeat centrifugation, the supernatant was subjected to LC–MS analysis. Chromatographic separation employed two complementary methods: Polar metabolites were separated on a Waters UPLC BEH Amide column (2.1 × 100 mm, 1.7 μm) using ammonium acetate/ammonium hydroxide in water (A) and acetonitrile (B); nonpolar metabolites were analysed using a Phenomenex C18 column (2.1 × 100 mm, 2.6 μm) with 0.01% acetic acid in water (A) and isopropanol/acetonitrile (1:1, v/v) (B). The autosampler temperature was maintained at 4°C, and 2 μL was injected per run. Mass spectrometry was conducted on an Orbitrap Exploris 120 instrument (Thermo Scientific) controlled by Xcalibur (v4.4). Key settings included sheath gas, 50 Arb; auxiliary gas, 15 Arb; capillary temperature, 320°C; MS1/MS2 resolutions: 60000 and 15 000; stepped NCE: 20, 30, 40 eV; spray voltage: ± 3.8 kV.

### 2.16 RNA Sequencing Analysis

Total RNA was isolated using TRIzol reagent, and RNA quality was assessed prior to library construction. Poly(A) + RNA was enriched, fragmented and reverse‐transcribed to generate sequencing libraries, which were sequenced on an Illumina platform to obtain paired‐end reads. After quality control, clean reads were aligned to the reference genome, and gene expression levels were quantified. Differentially expressed genes were identified using DESeq2 with a threshold of |log_2_FoldChange| > 1 and adjusted *p* < 0.05. Functional enrichment analyses were performed using Gene Ontology and KEGG databases.

### 2.17 Image Acquisition and Quantification

Images of the stained sections were analysed using the ImageJ software. Histological quantification was performed by an investigator blinded to group allocation to minimise observer bias. Image acquisition and analysis were conducted by different investigators.

### 2.18 Statistical Analysis

Continuous variables with normal distribution were expressed as mean ± SEM One‐way ANOVA (for single‐factor comparisons) or two‐way ANOVA (for two independent variables) were applied. All analyses were performed using GraphPad Prism 8.0 (GraphPad Software, San Diego, CA) and IBM SPSS Statistics 23.0 (IBM, Armonk, NY), with two‐tailed *p* value < 0.05 considered statistically significant.

## 3 Results

### 3.1 EE Was More Effective Than CE in Promoting Muscle Repair Following Acute Injury

**FIGURE 1:**
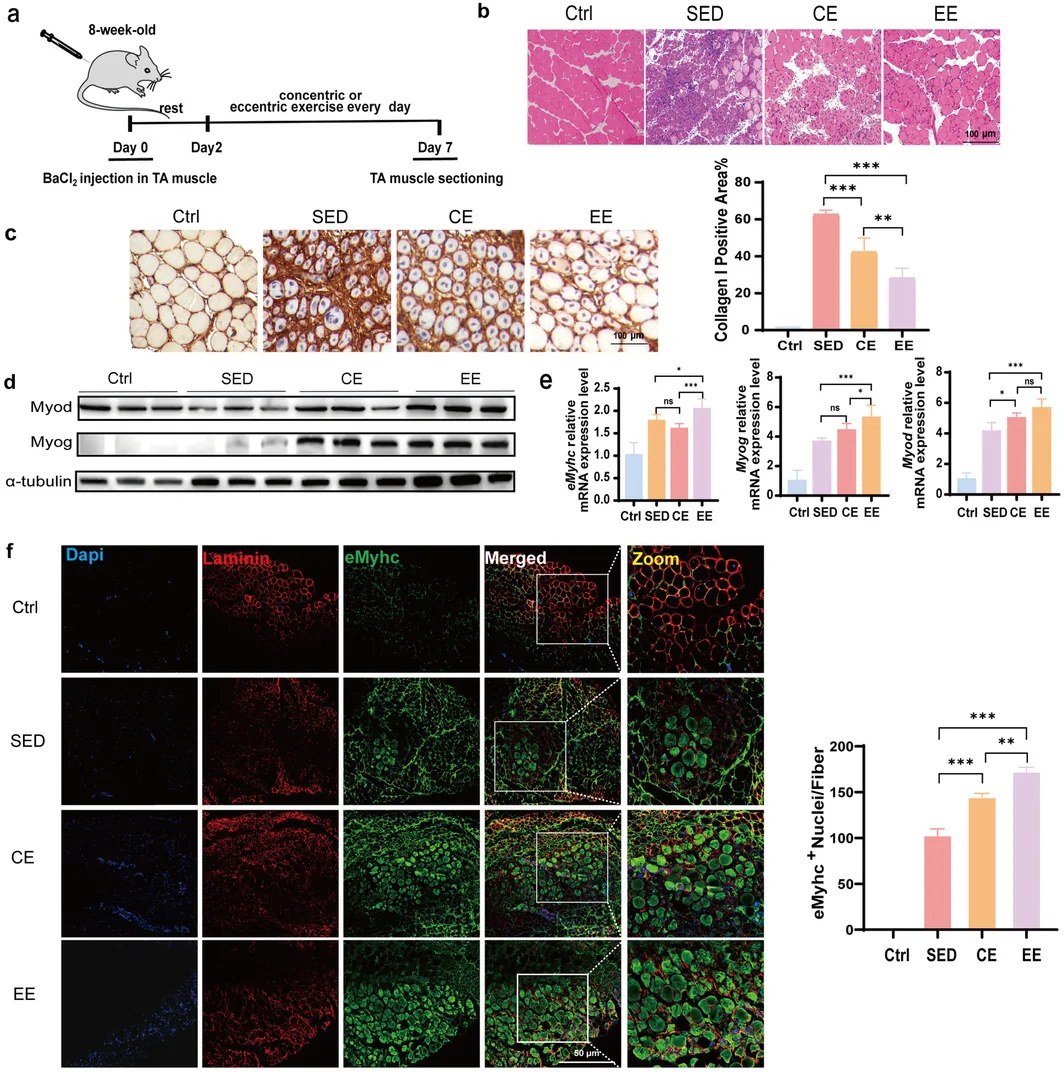
Eccentric exercise exerted more efficient effects than concentric exercise on promoting muscle repair following acute injury. (a) The schematic diagram of the experiments. (b) The representative images of H&E staining of TA muscle in mice from the undamaged group (Ctrl), sedentary group (SED), concentric exercise group (CE), and eccentric exercise group (EE). Scale bar, 100 μm. (c) The representative images of immunohistochemical staining for collagen Iα deposition in the Ctrl, SED, CE, and EE groups and quantitative analysis of collagen deposition between each group. Scale bar, 100 μm. (d) The protein expression levels of Myod and Myog, indicators of new muscle fibre formation, in different repair groups. (e) The qPCR analysis of mRNA expression levels of *Myog*, *Myod* and *eMyhc* in TA muscles of the Ctrl, SED, CE and EE groups. (f) Representative images of immunofluorescence staining for laminin (red) and eMyhc (green) in TA muscle sections of the Ctrl, SED, CE and EE groups, with quantitative analysis of eMyhc‐positive staining across groups. Nuclei were labelled with DAPI (blue). Scale bar, 50 μm. Data are presented as mean ± SEM *p < 0.05; **p < 0.01; ***p < 0.001 by one‐way ANOVA. *N* = 3 per group. CE, concentric exercise; EE, eccentric exercise; SED, sedentary; TA, tibialis anterior.

Based on preliminary time‐course experiments evaluating the regeneration kinetics, Day 7 post‐injury was determined as the optimal time point for assessing muscle repair (Figure S1a,b) [22]. Accordingly, to evaluate the beneficial effects of muscle contraction exercises on muscle repair, we subjected mice to 5 days of EE or CE on mice following barium chloride–induced muscle injury (Figure 1a). H&E staining revealed better organised morphological changes in the TA muscles of mice in the EE group compared with those in the CE group or SED group (Figure 1b). Immunohistochemical staining further demonstrated reduced collagen Iα deposition in the TA muscles of the EE group relative to the CE and SED groups (Figure 1c). As it was reported that *Myod*, *Myog* and *eMyhc* play a significant role in the process of muscle repair, we detected the expression levels of these markers. Western blot analysis indicated increased protein expression levels of Myod and Myog in the EE group compared with the CE and SED groups (Figure 1d and Figure S1c). Meanwhile, qPCR analysis revealed elevated mRNA expression levels of *Myod*, *Myog* and *eMyhc* in the EE group relative to the CE and SED groups (Figure 1e). We evaluated the maximal tetanic force of the EDL muscle in the EE group, which was higher than that in the CE and SED groups (Figure S1d). Immunofluorescent staining demonstrated more eMyhc‐positive fibres in EE group compared with the CE and SED groups, which indicated that more newborn fibres formed during muscle repair following EE (Figure 1f). Taken together, these findings suggest that EE could exert more efficient effects than CE on promoting muscle repair following acute injury.

### 3.2 EE Promoted More EV Secretion From Skeletal Muscle Than CE During Muscle Repair

**FIGURE 2:**
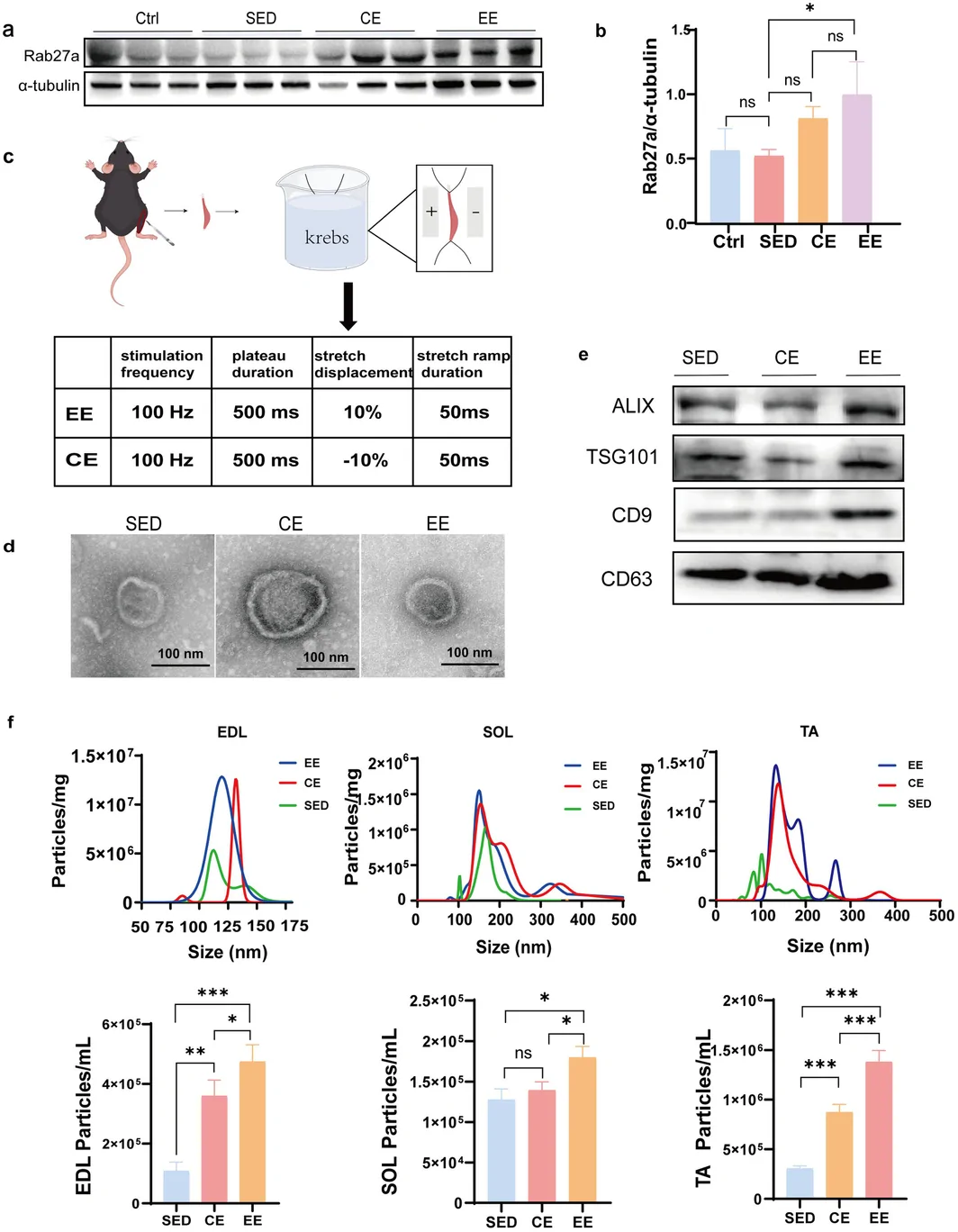
Eccentric exercise promoted more extracellular vesicles secretion from skeletal muscle than concentric exercise during muscle repair. (a) The protein expression levels of Rab27a in different repair groups. (b) Quantitative analysis of Rab27a expression between each group in (a). (c) The schematic diagram of Aurora Scientific instrument in vitro simulation. (d) Representative electron microscopy images of mEVs. Scale bar, 100 nm. (e) The protein expression levels of CD9, CD63, TSG101, ALIX, indicators of mEVs, in SED, CE and EE groups. (f) Particle size distribution of mEVs secreted by EDL and SOL in vitro simulating concentric and eccentric exercise. Particle size distribution of mEVs extracted from TA muscle tissue of exercised mice by enzymatic digestion. Data are presented as mean ± SEM *p < 0.05; **p < 0.01; ***p < 0.001 by one‐way ANOVA. For mEV isolation, *N* = 10 mice per group were used to ensure sufficient yield. For all other in vivo experiments, *N* = 3 mice per group were included. CE, concentric exercise; EDL, extensor digitorum longus; EE, eccentric exercise; mEVs, muscle‐derived extracellular vesicles; Sed, sedentary; SOL, soleus; TA, tibialis anterior.

Previous studies [11] have shown that muscle exercise can induce skeletal muscle to release EVs. We hypothesised that the superior efficacy of EE over CE in promoting muscle repair could be explained by the differences in mEVs between the two exercise modalities. Thus, we investigated mEV release from skeletal muscle induced by EE and CE. Western blot analysis indicated an increased protein expression level of Rab27a in the EE group than that in the CE and SED groups (Figure 2a,b). Meanwhile, qPCR analysis showed that the mRNA expression levels of *Rab27a* and *Rab27b* were elevated in the EE group compared with the CE SED groups (Figure S2a). We further performed ex vivo EE and CE on isolated muscle using the Aurora Scientific muscle testing system and collected the mEVs released from the muscle for subsequent analyses (Figure 2c). TEM confirmed that the isolated EVs appeared as round vesicles (Figure 2d). We also observed elevated protein expression levels of CD63, ALIX, TSG101 and CD9 in EVs isolated from the EE group than those from the CE and SED groups (Figure 2e). NTA indicated that the number of mEVs secreted from SOL, EDL and TA muscles during ex vivo contraction was higher in the EE group than that in the CE and SED groups (Figure 2f). In summary, these data indicated that EE promoted more mEVs secretion from skeletal muscle than CE during muscle repair.

### 3.3 Blockage of mEV Secretion From Skeletal Muscle Using GW4869 Abolished the Beneficial Effects of EE on Muscle Repair

**FIGURE 3:**
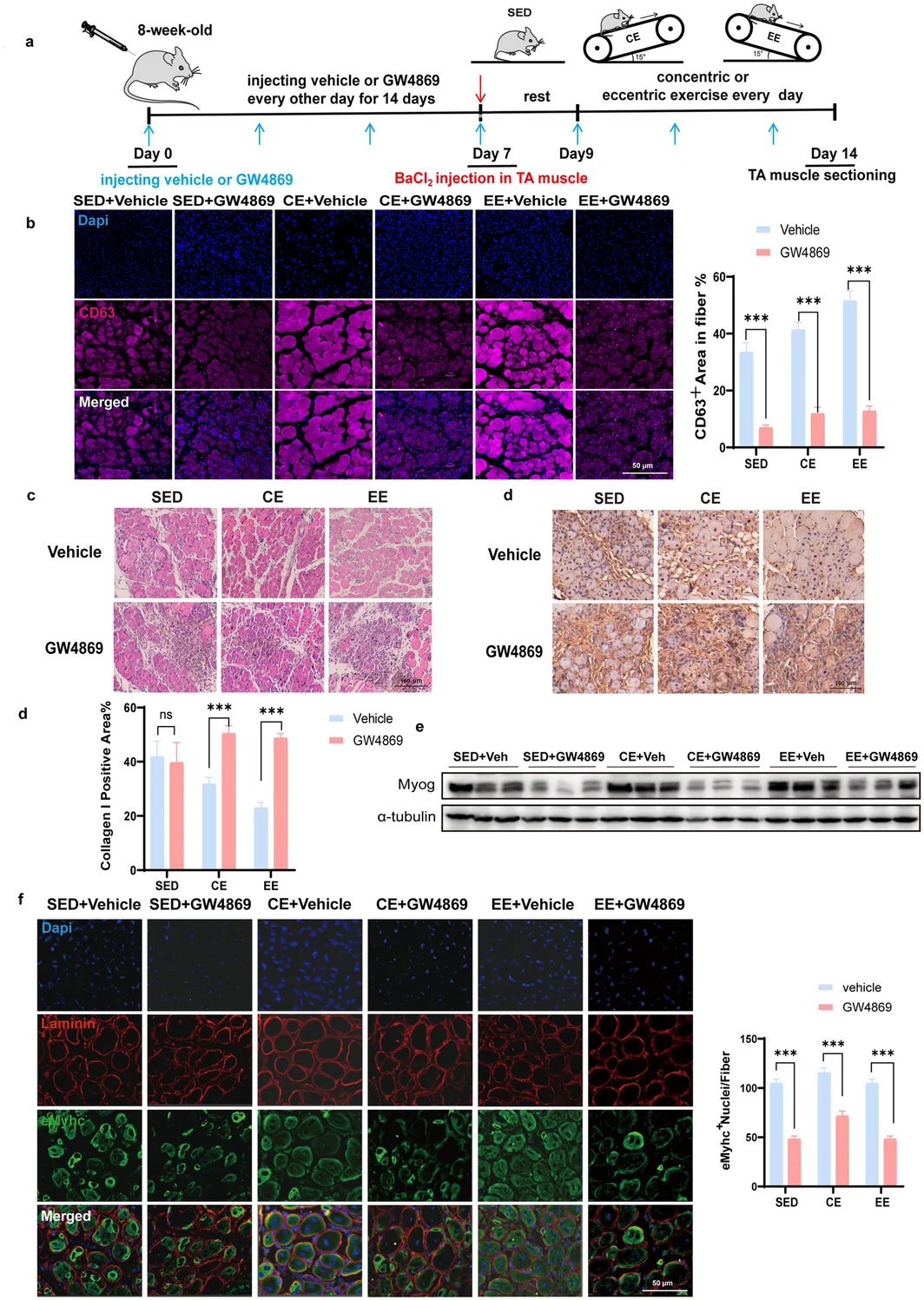
Blockage of mEV secretion from skeletal muscle using GW4869 abolished the beneficial effects on muscle repair by eccentric exercise. (a) The schematic diagram of the experiments. (b) The representative images of immunofluorescent staining for CD63 in TA muscle sections treated with GW4869 or vehicle in the SED, CE and EE groups and quantitative analysis of CD63‐positive staining among each group. Scale bar, 50 μm. (c) The representative images of H&E staining of TA muscle in mice from the GW4869 group and the vehicle group. Scale bar, 100 μm. (d) The representative images of immunohistochemical staining for collagen Iα deposition in GW4869 group and vehicle group and quantitative analysis of collagen deposition in immunohistochemical staining. Scale bar, 100 μm. (e) The protein expression levels of Myog, indicator of new muscle fibre formation, in the GW4869 group and the vehicle group. (f) Representative images of immunofluorescence staining for laminin (red) and emyhc (green) in TA muscle sections treated with GW4869 or vehicle in SED, CE and EE groups. Nuclei were labelled with DAPI (blue). Scale bar, 50 μm. Data are presented as mean ± SEM *p < 0.05; **p < 0.01; ***p < 0.001 by two‐way ANOVA. *N* = 3 per group. CE, concentric exercise; EE, eccentric exercise; SED, sedentary; TA, tibialis anterior.

To investigate the functional role of mEVs secreted from skeletal muscle in muscle repair during exercise, we used GW4869, an mEV inhibitor, to block mEV production in skeletal muscle (Figure 3a). Intramuscular administration of GW4869 effectively inhibited mEV production in skeletal muscle during exercise, as evidenced by a marked reduction in the CD63‐positive staining area compared with vehicle‐treated controls across the SED, CE and EE groups (Figure 3b). Consistent with this observation, both mRNA and protein expression levels of Rab27a and Rab27b were also decreased relative to vehicle‐treated controls in all groups (Figure S3a). Histological analyses further revealed more disorganised morphology by H&E staining (Figure 3c) and increased collagen Iα deposition by immunohistochemical staining (Figure 3d) in GW4869‐treated muscles across the SED, CE and EE groups. In addition, the mRNA expression levels of *Myod*, *Myog* and *eMyhc* (Figure S3b), as well as the protein expression level of Myog, were decreased in GW4869‐treated muscles across the SED, CE and EE groups (Figure 3e and Figure S3c). Immunofluorescent staining also demonstrated less eMyhc‐positive staining fibres in GW4869‐treated muscles (Figure 3f). Together, these data suggested that GW4869‐mediated inhibition of mEV production impairs the muscle repair process. Interestingly, no significant differences were observed in muscle repair–related parameters between the CE and EE groups after GW4869 injection. This suggests that the mEVs play an important role in muscle repair and that inhibiting mEV production using GW4869 could abolish the beneficial effects of EE on muscle repair.

### 3.4 The mEVs Induced by EE Exerted More Beneficial Effects on Promoting Muscle Repair Than Those Generated by CE

**FIGURE 4:**
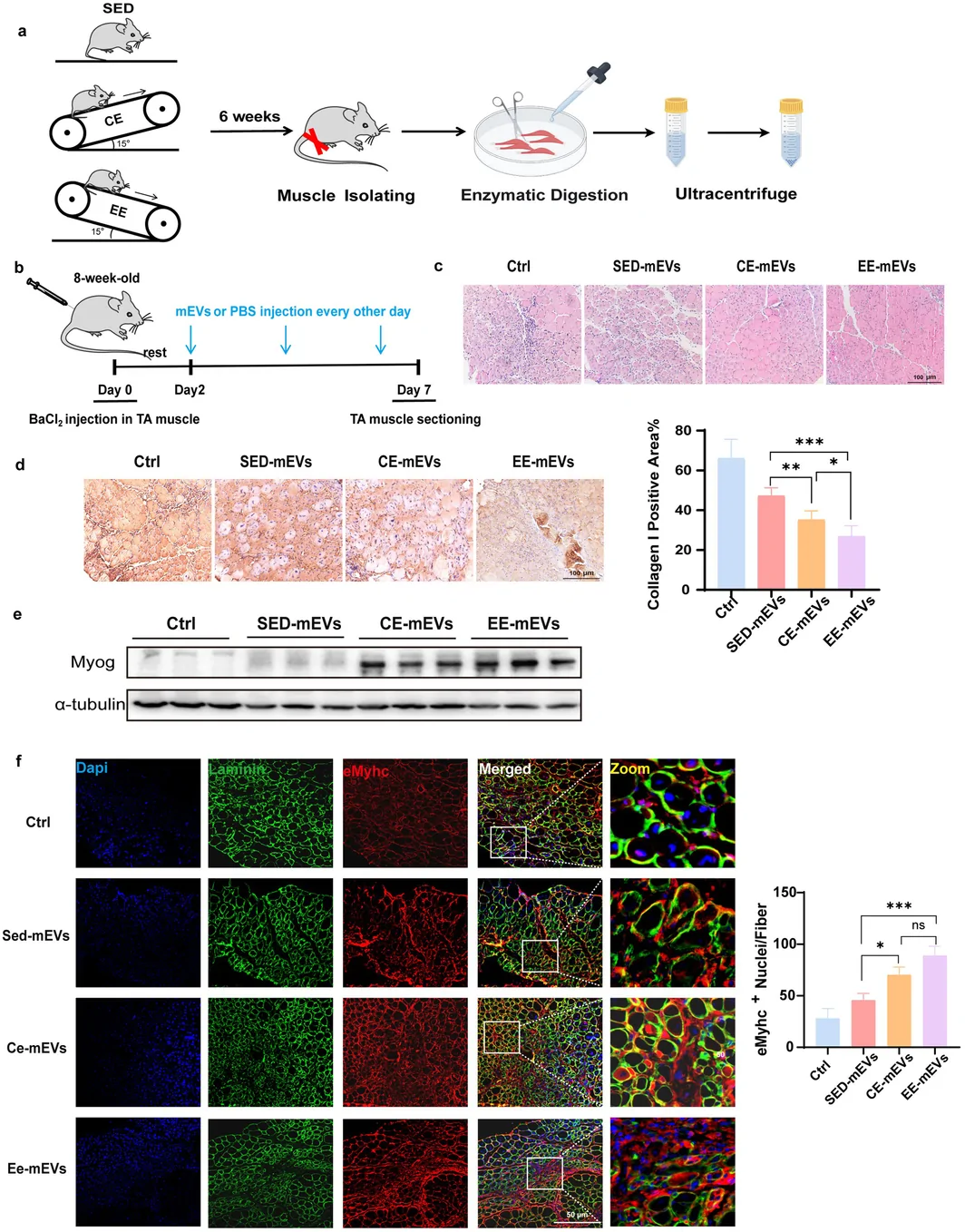
The mEVs induced by eccentric exercise exerted more beneficial effects on promoting muscle repair than those generated by concentric exercise. (a) The schematic diagram of the experiments of collecting mEVs in the SED, CE and EE groups. (b) The schematic diagram of the experiments of injecting PBS or mEVs to mice after muscle injury. (c) The representative images of HE staining of TA muscle in mice from Ctrl, SED‐mEVs, CE‐mEVs and EE‐mEVs. Scale bar, 100 μm. (d) The representative images of immunohistochemical staining for collagen Iα deposition in the Ctrl, SED‐mEV, CE‐mEV and EE‐mEV groups and quantitative analysis of collagen deposition among each group. Scale bar, 100 μm. (e) The western blotting analysis for the protein expression levels of Myod and Myog in the Ctrl, SED‐mEV, CE‐mEV and EE‐mEV groups. (f) Representative images of immunofluorescence staining for laminin (red) and emyhc (green) in TA muscle sections of the Ctrl, SED‐mEVs, CE‐mEV and EE‐mEV groups, with quantitative analysis of emyhc positive staining across groups. Nuclei were labelled with DAPI (blue). Scale bar, 50 μm. Data are presented as mean ± SEM *p < 0.05; **p < 0.01; ***p < 0.001 by one‐way ANOVA. *N* = 3 per group. CE‐mEVs, mEVs derived from muscle of mice in the concentric exercise group; EE‐mEVs, mEVs derived from muscle of mice in the eccentric exercise group; SED‐mEVs, mEVs derived from muscle of mice in the resting group; TA, tibialis anterior.

To further confirm the functional role of mEVs in muscle repair during exercise, we isolated the mEVs released from skeletal muscle during SED, CE and EE (Figure 4a) and intramuscularly injected them into mice during muscle repair (Figure 4b). TEM confirmed that isolated mEVs appeared as round vesicles (Figure S4a). We found that mEVs can enter muscle SCs by labelling EVs with PKH26 (Figure S4b). H&E staining revealed better organised morphological changes in the repaired muscle tissue of mice injected with mEVs from EE (EE‐mEVs group) compared with those injected with mEVs from CE (CE‐mEV group) or SED (SED‐mEV group) (Figure 4c). We also found less collagen Iα deposition in repaired muscle by immunohistochemical staining in the EE‐mEV group compared with the CE‐mEV and SED‐mEV groups (Figure 4d). Western blot analysis indicated an increase in protein expression levels of Myog in the EE‐mEV group than that in the CE‐mEV and SED‐mEV groups (Figure 4e and Figure S4b). Meanwhile, qPCR analysis revealed the mRNA expression levels of *Myod*, *Myog* and *eMyhc* were elevated in the EE‐mEV group compared with those in the CE‐mEV and SED‐mEV groups (Figure S4c). Subsequent immunofluorescent staining demonstrated that more eMyhc‐positive fibres in the EE‐mEV group compared with that in the CE‐mEV and SED‐mEV groups, which indicated that more newborn fibres formed during muscle repair following EE (Figure 4f). Taken together, these data indicated that exogenous supplementation of EE‐mEVs exerted more beneficial effects on promoting muscle repair than CE‐mEVs.

### 3.5 Lipid Metabolites Enriched in EE‐mEVs and EE Promoted Lipid Metabolism in Skeletal Muscle

**FIGURE 5:**
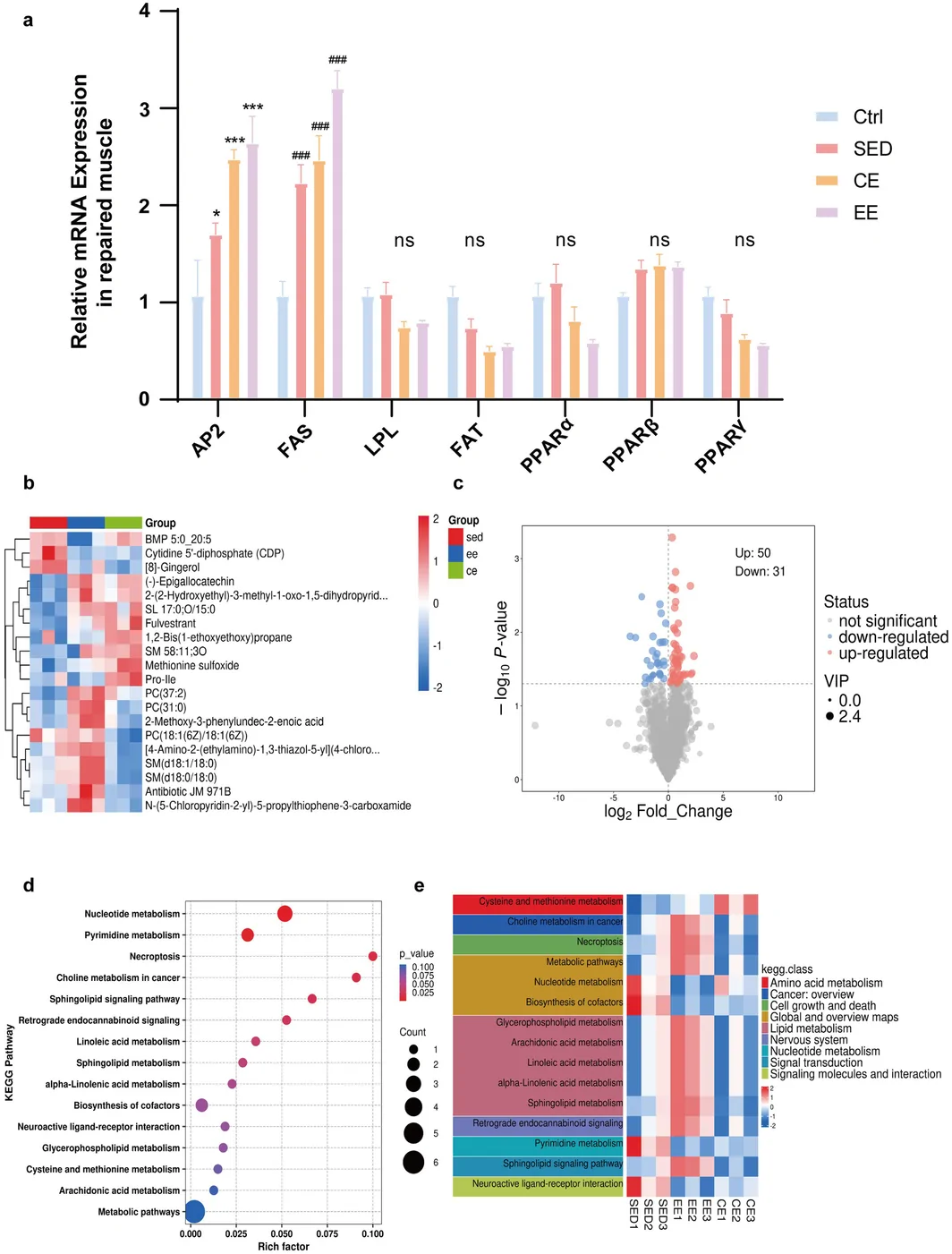
Lipid metabolites enriched in EE‐mEVs and EE promoted lipid metabolism in skeletal muscle. (a) The qPCR analysis of mRNA expression levels of *AP2*, *FAS*, *LPL*, *FAT*, *PPARα*, *PPARβ* and *PPARγ* in TA muscles of the Ctrl, SED, CE and EE groups. (b) Heat map showing differences in metabolites contained in mEVs from the SED, CE and EE groups. (c) Volcano plot showing that muscle metabolite PC (37:2) is upregulated during EE. (d–e) KEGG enrichment analysis bubble chart (C) and heat map (D) both show that lipid metabolism is active during EE. Notes: Data are presented as mean ± SEM *p < 0.05; **p < 0.01; ***p < 0.001 by one‐way ANOVA. *N* = 5 for per group for metabolomics. CE, concentric exercise; EE, eccentric exercise; SED, sedentary; TA, tibialis anterior.

Previous studies have shown that both CE and EE significantly influence metabolomics, particularly lipid metabolites [17]. To investigate this, we performed RT‐qPCR to detect lipid metabolism‐related markers, including AP2, FAS, LPL, FAT, PPARα, PPARβ and PPARγ (Figure 5a). The results indicated that the activity trends of AP2 and FAS were consistent with the preceding analysis. It has been established that AP2 and FAS primarily function in the synthesis of fatty acids and sterols. To further explore the differences among EE‐mEVs, CE‐mEVs and SED‐mEVs, we performed non‐targeted metabolomics analysis of mEVs. Compared with the CE and SED groups, lipid metabolites were most enriched among the upregulated metabolites in the EE group (Figure 5b,c), consistent with the KEGG analysis (Figure 5d,e). KEGG enrichment analysis further showed that lipid metabolism of EE‐mEVs was significantly more active than that of CE‐mEVs and SED‐mEVs. Consequently, it can be inferred that the enrichment of lipid metabolites in EE‐mEVs promotes the synthesis of fatty acids and sterols, thereby facilitating muscle injury recovery.

### 3.6 EE‐mEVs More Effectively Promoted Myoblast Differentiation Than CE‐mEVs or SED‐mEVs

**FIGURE 6:**
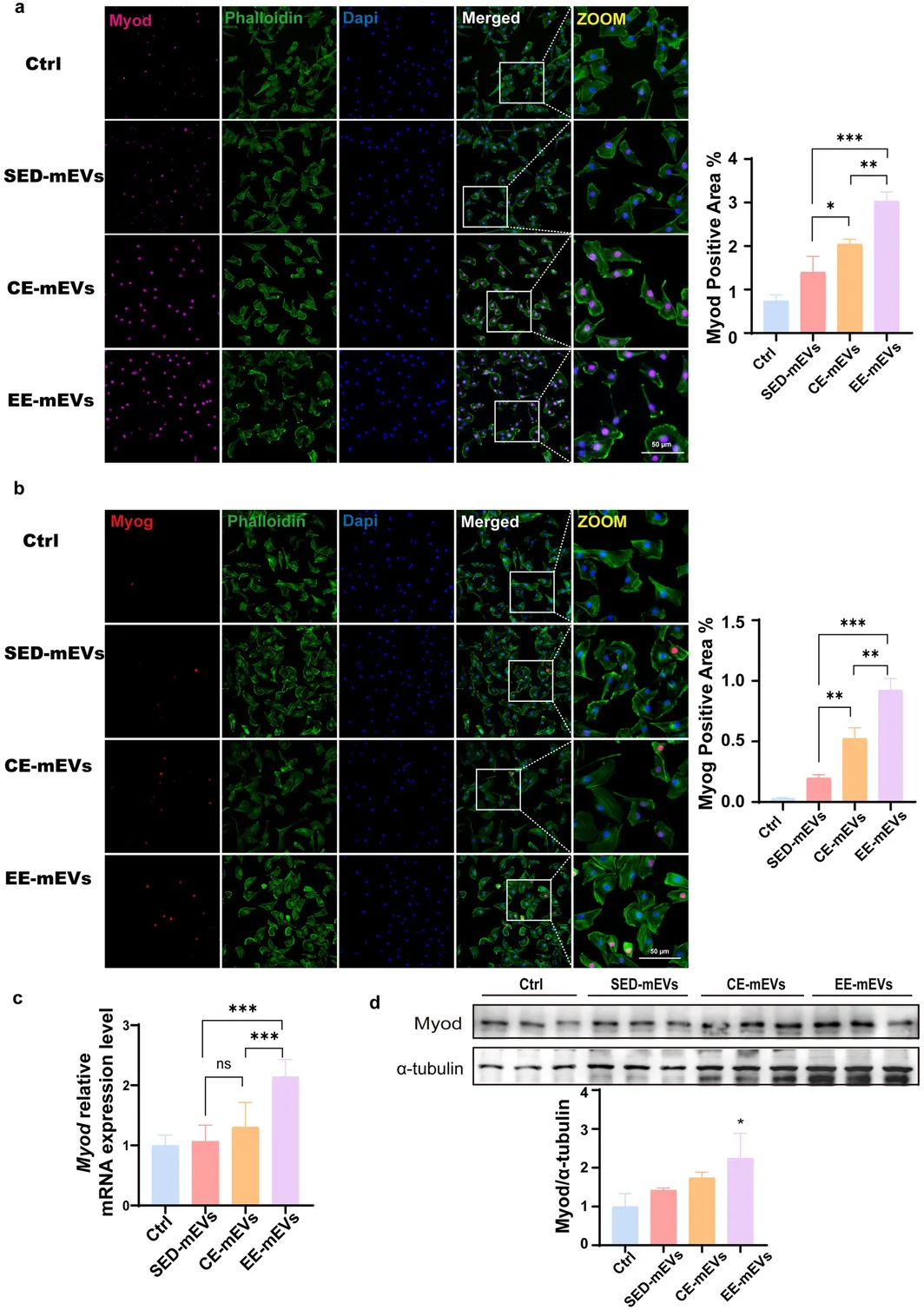
EE‐mEVs exhibited higher degree effects than CE‐mEVs or SED‐mEVs on promoting myoblast differentiation. (a) Left panel, representative images of immunofluorescence staining for Myod (red) and phalloidin (green) of the Ctrl, SED‐mEV, CE‐mEV and EE‐mEV groups. Nuclei were labelled with DAPI (blue). Scale bar, 50 μm. Right panel, quantitative analysis of Myod‐positive staining among each group. (b) Left panel, representative images of immunofluorescence staining for Myog (red) and phalloidin (green) of the Ctrl, SED‐mEV, ce‐mEV and EE‐mEV groups. Nuclei were labelled with DAPI (blue). Scale bar, 50 μm. Right panel, quantitative analysis of Myog‐positive staining among each group. (c) The qPCR analysis for the mRNA expression levels of *Myod* of the Ctrl, SED‐mEV, CE‐mEV and EE‐mEV groups. (d) The western blotting analysis for the protein expression levels of Myod in the Ctrl, SED‐mEV, CE‐mEV and EE‐mEV groups. Data are presented as mean ± SEM *N* = 3 per group. *p < 0.05; **p < 0.01; ***p < 0.001 by one‐way ANOVA. CE‐mEVs, extracellular vesicles derived from muscle of mice in the concentric exercise group; EE‐mEVs, extracellular vesicles derived from muscle of mice in the eccentric exercise group; SED‐mEVs, extracellular vesicles derived from muscle of mice in the resting group.

To further investigate the effects of mEVs from different exercise sources on myoblast differentiation, we added previously extracted muscle‐derived EVs (SED‐mEVs, CE‐mEVs, EE‐mEVs) to the culture medium and co‐cultured them with C2C12 myoblasts. We found that mEVs can enter myoblasts by labelling EVs with PKH26 (Figure S5a). Immunofluorescent staining demonstrated more Myod‐positive cells in the EE‐mEV group than in the CE‐mEV and SED‐mEV groups, indicating enhanced differentiation of C2C12 myoblasts upon the addition of EE‐mEVs to the co‐culture (Figure 6a). Similarly, we observed more Myog‐positive cells in the EE‐mEVs group than in the CE‐mEV and SED‐mEV groups, further supporting enhanced C2C12 myoblast differentiation following EE‐mEV treatment (Figure 6b). Consistently, qPCR analysis revealed that the mRNA expression level of *Myod* was elevated in the EE‐mEV group compared with the CE‐mEV and SED‐mEV groups (Figure 6c). Western blot analysis also indicated an increase in the protein expression level of Myod in the EE‐mEV group compared with the CE‐mEV and SED‐mEV groups (Figure 6d). In summary, these data indicate that treatment with EE‐mEVs significantly promotes C2C12 myoblast differentiation compared with CE‐mEVs or SED‐mEVs.

### 3.7 Blockage of the Lipid Metabolism in Muscle Abolished the Beneficial Effects of CE/EE‐mEVs on Muscle Repair

**FIGURE 7:**
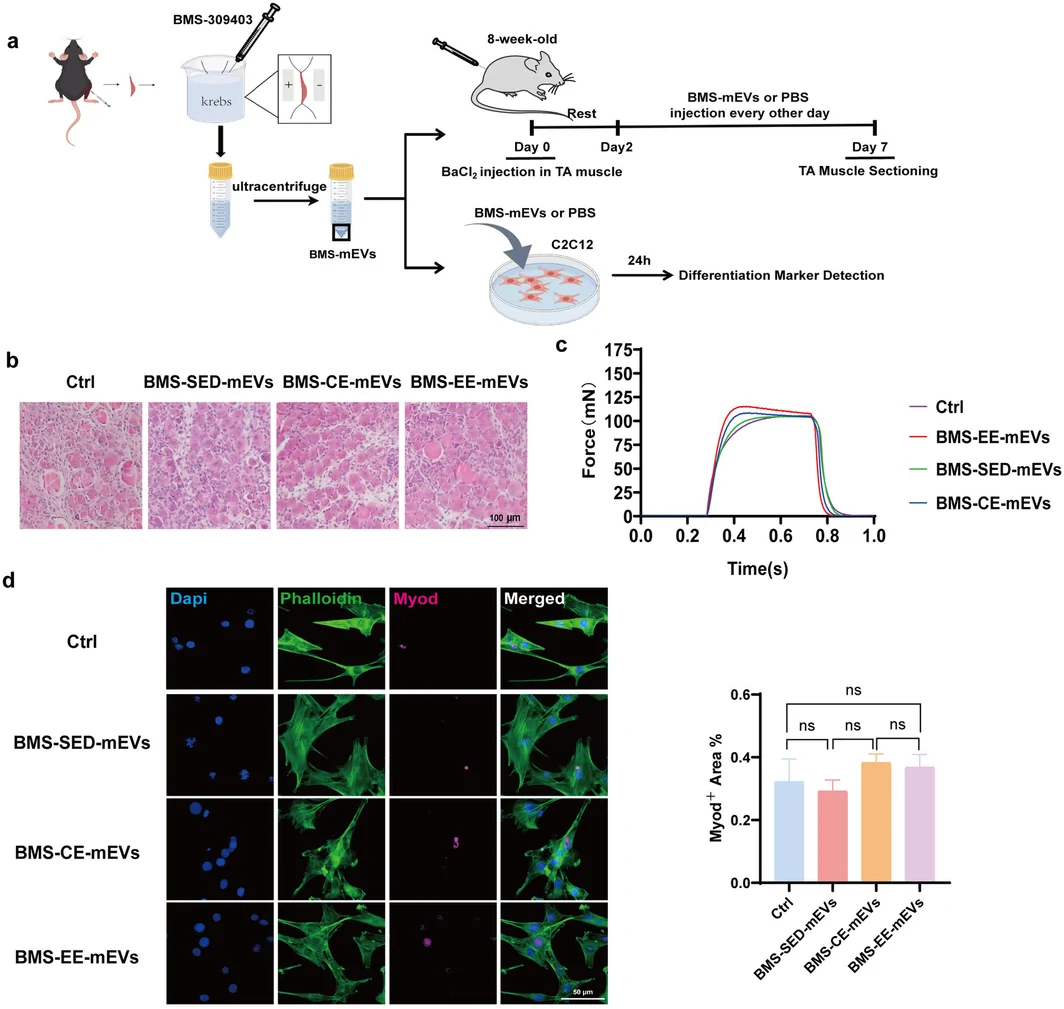
Blockage of the lipid metabolism in muscle abolished the beneficial effects of CE/EE‐mEVs on muscle repair. (a) The schematic diagram of the experiments. (b) The representative images of HE staining of TA muscle in mice from Ctrl, BMS‐SED‐mEVs, BMS‐CE‐mEVs and BMS‐EE‐mEVs. Scale bar, 100 μm. (c) The representative curve for tetanic force generated during tetanic stimulation in EDL from the Ctrl, BMS‐SED‐mEV, BMS‐ce‐mEV and BMS‐EE‐mEV groups. (d) Left panel, representative images of immunofluorescence staining for Myod (red) and phalloidin (green) of the Ctrl, BMS‐SED‐mEV, BMS‐CE‐mEV and BMS‐EE‐mEV groups. Nuclei were labelled with DAPI (blue). Scale bar, 50 μm. Right panel, quantitative analysis of Myod‐positive staining among each group. Data are presented as mean ± SEM *p < 0.05; **p < 0.01; ***p < 0.001 by one‐way ANOVA. For BMS‐mEVs isolation, *N* = 10 mice per group were used to ensure sufficient yield. For all other experiments, *N* = 3 mice per group were included.

AP2 and FAS were identified as the key regulators responsible for enhanced lipid metabolism in muscle in the CE and EE groups (Figure 5a). Furthermore, western blot analyses confirmed that only AP2 protein levels were increased in the CE and EE groups compared with those in the SED group (Figure S6a), which indicate that AP2 was mainly responsible for increased synthesis of fatty acids and sterols in muscle after exercise. Therefore, we used BMS‐309403, an AP2 inhibitor, to block the enhanced lipid metabolism in the isolated muscle during ex vivo CE/EE training. Subsequently, we extracted mEVs from the muscle and then intramuscularly injected them into mice during muscle repair or co‐cultured them with C2C12 cells (Figure 7a). We observed no significant differences in both morphological changes by H&E staining (Figure 7b) and muscle function (Figure 7c) among the Ctrl, BMS‐SED‐mEV, BMS‐CE‐mEV and BMS‐EE‐mEV groups. In consistent with above in vivo data, we also found that there were no significant differences in Myod expression level of C2C12 cells by immunofluorescence among the Ctrl, BMS‐SED‐mEV, BMS‐CE‐mEV and BMS‐EE‐mEV groups (Figure 7d). Taken together, we demonstrated that the blockage of the lipid metabolism in muscle could abolish the beneficial effects of CE/EE‐mEVs on muscle repair.

### 3.8 Lipid Metabolites Enriched in mEVs Contributed to Muscle Repair Potentially Through Upregulating Energy‐Sensing Genes Associated With Fatty Acid Metabolism in Myoblasts

**FIGURE 8:**
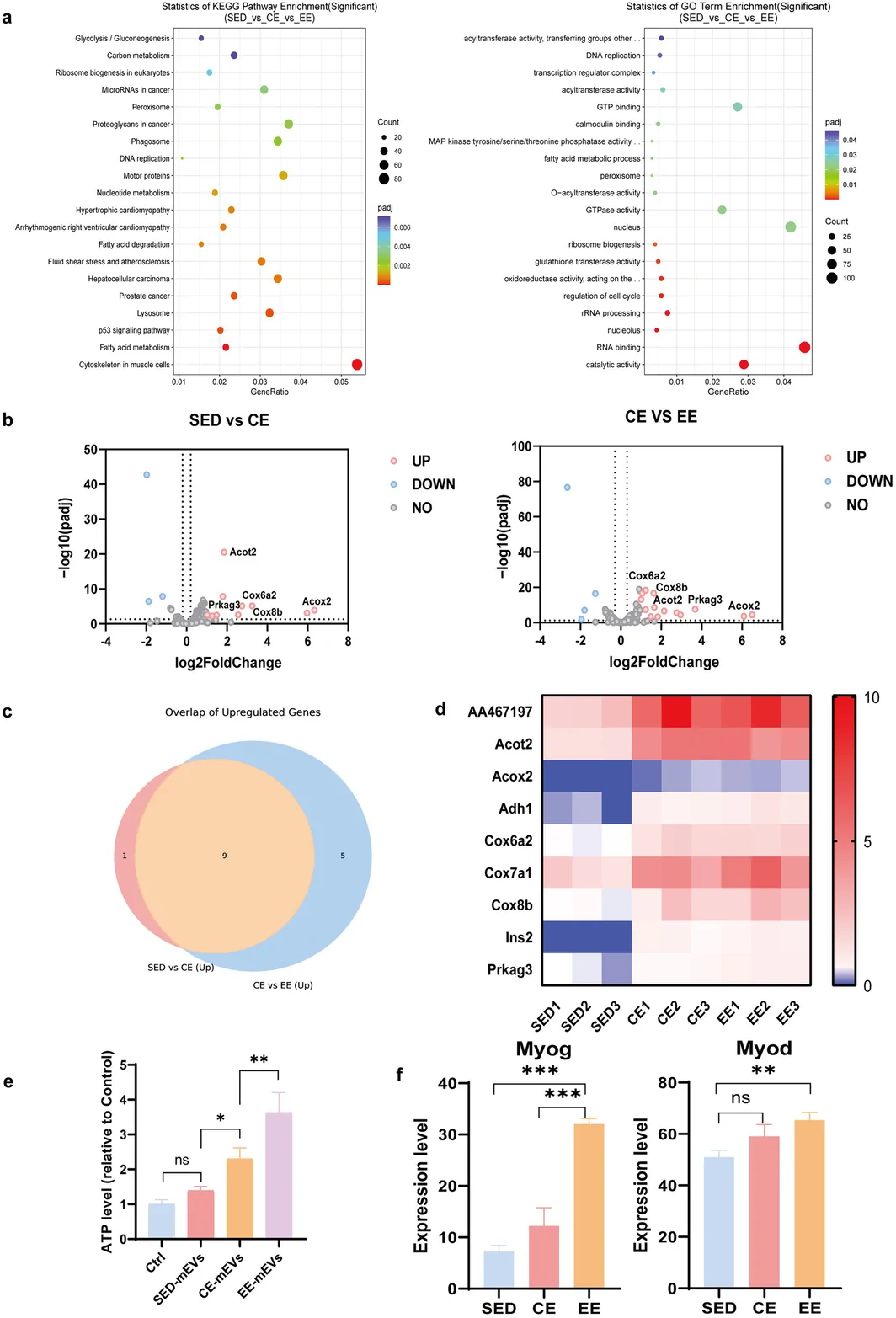
Lipid metabolites enriched in mEVs contributed to muscle repair potentially through upregulating energy‐sensing genes associated with fatty acid metabolism in myoblasts. (a) Left panel, KEGG pathway enrichment analysis of differentially expressed genes among the SED, CE and EE groups. Right panel, Gene Ontology (GO) enrichment analysis of differentially expressed genes following exercise‐derived mEV treatment. (b) Left panel, volcano plot of differentially expressed genes (DEGs) between the SED and CE groups. Right panel, volcano plot of DEGs between the CE and EE groups. (c) Overlap of upregulated genes between SED versus CE and CE versus EE comparisons. (d) Heat map of the expression of the genes overlapped in (d). (e) ATP levels in C2C12 cells after 24 h incubation with the Ctrl, SED‐EV, CE‐EV and EE‐EV groups. (f) The expression of myogenic markers Myog and Myod. Data are presented as mean ± SEM *N* = 3 per group. *p < 0.05; **p < 0.01; ***p < 0.001 by one‐way ANOVA.

To explore the molecular mechanism for lipid metabolites‐enriched mEVs promoting muscle repair, we treated C2C12 cells with SED‐mEVs, CE‐mEVs and EE‐mEVs for 24 h and subsequently performed the RNA‐sequencing. Based on the transcriptomic analysis results, KEGG and GO analysis enriched in fatty acid metabolism pathway (Figure 8a). Furthermore, the analysis of differentially expressed genes involved in fatty acid metabolism revealed a sequential increase in the expression of nine genes across the SED, CE and EE groups (Figure 8b,c). Notably, among these candidate genes, *Prkag3* has been previously verified to promote muscle injury repair [23], whereas *Cox7a1*, *Cox6a2* and *Cox8b* are energy‐sensing genes highly expressed in skeletal muscle (Figure 8d). Given these findings, we assessed ATP levels and found that ATP level in the EE‐mEV group was higher than those in the CE‐mEV and SED‐mEV groups (Figure 8e). Meanwhile, we also found increased expression levels of myogenic markers *Myog* and *Myod* in the EE‐mEV group compared with the CE‐mEV and SED‐mEV groups. Taken together, these data indicated that lipid metabolites enriched in mEVs contributed to muscle repair potentially through upregulating energy‐sensing genes associated with fatty acid metabolism in myoblasts.

To further assess the contribution of specific lipid components, we performed in vitro supplementation experiments using PI (18:1/18:1), a lipid highly enriched in EE‐mEVs. PI supplementation promoted AMPK activation, as evidenced by an increased p‐AMPK/AMPK ratio (Figure S7a). Consistently, PI treatment also significantly enhanced the myogenic differentiation of myoblasts, as indicated by increased MyoD expression, and partially rescued the pro‐myogenic capacity of both SED‐mEVs and CE‐mEVs (Figure S7b). To further evaluate the functional role of AMPK, we applied the pharmacological inhibitor Compound C. Inhibition of AMPK signalling significantly attenuated the pro‐myogenic effects, supporting the involvement of AMPK in mediating the observed regenerative responses (Figure S7c,d). Collectively, these results provide functional evidence that PI (18:1/18:1) contributes to the enhanced regenerative effects of EE‐mEVs. Mitochondrial membrane potential was also assessed using JC‐1 staining. EE‐mEV treatment increased mitochondrial membrane potential compared with the SED‐mEV and CE‐mEV groups, suggesting enhanced mitochondrial function (Figure S8).

## 4 Discussion

Skeletal muscle injuries, including quadriceps injuries and hamstring injuries, are extremely common among athletes and the general populations in daily life and various sporting events [24]. Physical exercise is widely recognised as an efficient approach to promote muscle repair and improve muscle function after injury [25]. It has been reported that voluntary wheel running induces myogenic factors related to muscle repair, in which 4 weeks of running were followed by a 2‐week resting phase [26]. Researchers also demonstrated that a running protocol consisting of a 5‐min warm‐up at 5 m/min, followed by a 42‐min run at 60% of maximum running speed and a 5‐min cool‐down at 5 m/min every 2 days, boosted muscle regeneration in aged mice [27]. Consistently, we used the BaCl_2_‐induced muscle injury model because it reproducibly induces myofibre necrosis while preserving the basal lamina and SC niche, allowing synchronised muscle regeneration [28]. This model is also clinically relevant, as it recapitulates key histopathological features of common muscle injuries with intact structural scaffolds [29]. Our study also proved that 5‐day treadmill running exerted beneficial effects on muscle regeneration after injury. For muscle physical exercise, there are three types of exercise protocols including isometric exercise, CE and EE [30]. Previous reports have indicated that EE is more beneficial for muscle repair than CE [31], and EE reduced the incidence of hamstring injury by 56.8% to 70.0% [32]. Consistent with these findings, we also observed that EE exerted greater effects than CE in promoting muscle repair following acute injury.

Recent studies have extensively investigated the mechanisms by which exercise facilitates muscle damage repair. On the one hand, exercise directly promotes SC self‐renewal and differentiation [33]. On the other hand, exercise can induce skeletal muscle to release myokines including irisin, interleukin‐15 (IL‐15) and musclin participating in muscle regeneration. Irisin secreted from skeletal muscle in response to exercise plays a significant role in promoting muscle growth, enhancing muscle regeneration, activating MuSCs and potentially regulating metabolism [34, 35]. Exercise‐induced activation of p38γ in muscle leads to the production of IL‐15 and has protective functions on skeletal muscle [36]. Exercise also induces skeletal muscle to release Musclin, which subsequently upregulates FILIP1L to reduce the number of FAPs through AKT‐FoxO3a signalling, thereby promoting muscle regeneration [8]. Previous studies have shown that skeletal muscle can release EVs during exercise [37]. However, whether mEVs during contraction exercise participate in muscle repair is still unclear. In our study, we found that exercise induces skeletal muscle to release EVs and that EE promotes more EV secretion than CE during muscle repair. Furthermore, we demonstrated that administration of exogenous EVs from exercised muscle could promote muscle repair and new fibre generation, whereas blockage of EV secretion from skeletal muscle using GW4869 abolished the beneficial effects of exercise on muscle repair. Therefore, we proved that exercise could induce skeletal muscle to secrete EVs to promote muscle repair after injury. Interestingly, building on the observed differences in EV size distribution among the SED, CE and EE groups, previous studies have shown that EVs can be broadly categorised into exosomes (~30–150 nm) and ectosomes (~150–1000 nm), which differ in both biogenesis and cargo composition [29]. Exosomes are enriched in lipid raft–associated lipids, vesicle biogenesis–related proteins and regulatory RNAs, particularly miRNAs, whereas ectosomes contain a more heterogeneous set of membrane, cytoskeletal and metabolic proteins that may more directly reflect the physiological status of the parent muscle fibres. Therefore, the altered EV size profile observed in TA muscle following EE may indicate shifts in EV subpopulation composition, potentially accompanied by distinct cargo characteristics and biological functions, which could contribute to the enhanced regenerative outcome. In addition, the secreted EV size distributions and abundance differed among muscle types, which reflect muscle‐specific EV populations rather than technical limitations. It may be due to the distinct muscle fibre composition in EDL (predominantly type II), SOL (predominantly type I) and TA (mixed) as well as their different response to exercise. However, the underlying mechanism is still needed to be explored in future.

mEVs are naturally occurring EVs that serve as essential carriers for a diverse array of biomolecules, including nucleic acids, proteins, lipids and metabolites [38], facilitating intercellular communication and playing a significant role in regenerative processes. Growing evidence supports their therapeutic potential in mitigating muscle damage. For instance, mEVs exhibit high levels of miR‐4331‐3p, which enhances myoblast proliferation, suggesting that S mEVs may orchestrate muscle regeneration through selective packaging of regulatory miRNAs [39]. Beyond nucleic acids, recent attention has turned to metabolites as critical signalling entities influencing cellular functions. Lipid metabolites, in particular, are emerging as key modulators of protein activity and intracellular signalling cascades [40]. Our metabolomic analysis identified phosphatidylcholines (PCs) and sphingomyelins (SMs) as the primary lipid classes enriched in EE‐mEVs. PCs are critical substrates for membrane remodelling, a process essential for myoblast fusion and muscle fibre growth [41]. Meanwhile, SMs are key components of lipid rafts that orchestrate myogenic signalling pathways involved in differentiation [42]. Thus, EE‐mEVs may facilitate regeneration, at least in part, through delivery of these lipids to support both structural membrane repair and pro‐regenerative signalling. RNA‐seq analysis further revealed that exercise‐derived mEVs are associated with the activation of fatty acid metabolism–related pathways in myoblasts. The increased expression of mitochondrial genes, including COX7A1 and COX6A2, suggests enhanced oxidative metabolism, a process known to support myogenic differentiation and tissue regeneration. Moreover, the upregulation of energy‐sensing genes such as PRKAG3 implies the involvement of AMPK‐related metabolic signalling. Together, these metabolic adaptations may contribute to the pro‐regenerative effects of EE/CE‐mEVs during muscle repair.

Although exercise facilitates muscle recovery post‐injury, improper management of intensity during EE may induce secondary damage [43]. These effects not only exacerbate tissue injury but also prolong recovery due to restricted movement. Our research highlights the potential of exogenous mEV supplementation as a standalone intervention to prevent these adverse outcomes. By mitigating exercise‐induced muscular damage, mEVs derived from muscle contractions may significantly shorten recovery time and improve rehabilitation efficacy. Consequently, the application of muscle contraction‐derived mEVs could eventually supplant conventional treatments, offering a novel therapeutic avenue for accelerated muscle recovery.

Although our findings indicate that exercise‐induced mEVs promote skeletal muscle repair, several limitations should be noted. First, although mEVs are released from skeletal muscle during exercise, other factors may also contribute to muscle regeneration and cannot be excluded. Second, our study used only male mice, and thus, the findings may not fully extrapolate to female mice. Third, the current study has not yet translated these findings into a therapeutic strategy. There are several challenges remain for clinical translation. These include scalable production with consistent quality, batch‐to‐batch variability and maintaining EV stability and bioactivity during storage and transport. In addition, efficient targeted delivery to injured skeletal muscle is difficult due to potential clearance and off‐target distribution. Safety considerations, including immunogenicity and long‐term effects, also require further evaluation. Therefore, although exercise‐derived mEVs provide important mechanistic insights, their therapeutic application will require advances in EV standardisation, engineering and delivery. Fourth, although mitochondrial membrane potential was assessed in this study, more comprehensive analyses of mitochondrial function (e.g., oxygen consumption rate) would be required to fully characterise metabolic alterations. Nevertheless, the primary focus of this study is to investigate the role of exercise‐derived mEVs in skeletal muscle regeneration rather than to comprehensively dissect mitochondrial mechanisms. In addition, the lack of size‐fractionated EV analysis limits our ability to distinguish the relative contributions of specific EV subpopulations (e.g., exosomes versus microvesicles). Therefore, the observed effects may reflect the combined influence of both EV cargo composition and subpopulation distribution. Collectively, these limitations highlight the need for further studies to elucidate the biological functions of mEVs and to evaluate their potential for clinical application.

## Funding

This study was supported by the National Natural Science Foundation of China (92368201, 82272530, 82372459, 82502961), Science and Technology Cooperation Program (202308002), the Major Project of NSFC (81991514), the National Key Research and Development Project (2021YFA1201404), Jiangsu Province Medical Innovation Center of Orthopedic Surgery (CXZX202214), China Postdoctoral Science Foundation (2024T170409), Jiangsu Provincial Key Medical Center Foundation, Jiangsu Provincial Medical Outstanding Talent Foundation, Jiangsu Provincial Medical Youth Talent Foundation, Jiangsu Provincial Key Medical Talent Foundation and the Fundamental Research Funds for the Central Universities (14380493,14380494).

## Ethics Statement

The authors certify that they complied with the ethical guidelines for publishing in the Journal of Cachexia, Sarcopenia and Muscle. And all animal studies have been approved by the appropriate ethics committee.

## Conflicts of Interest

The authors declare no conflicts of interest.

## Supporting information


**Figure S1:** Eccentric exercise exerted more efficient effects than concentric exercise on promoting muscle repair following acute injury. (a) The representative images of H&E staining of TA muscle in mice from the undamaged group (Ctrl), sedentary group (SED), concentric exercise group (CE) and eccentric exercise group (EE) in Day 3, Day 5, Day 7 and Day 14. Scale bar, 100 μm. (b) Sections of the Ctrl, SED, CE and EE groups in Day 3, Day 5, Day 7 and Day 14, with quantitative analysis of eMyhc‐positive staining across groups. Nuclei were labelled with DAPI (blue). Scale bar, 50 μm. (c) Western blot analysis and quantification of Myod and Myog, which serve as indicators of new muscle fibre formation, were detected at the protein level in the SED, CE and EE groups. (d)Left panel, the representative curve for tetanic force generated during tetanic stimulation in EDL from the Ctrl, SED, CE and EE groups in Day 7. Right panel, the representative curve for tetanic force generated during tetanic stimulation in EDL from the Ctrl, SED, CE and EE groups in Day 14. Data are presented as mean ± SEM *p < 0.05; **p < 0.01; ***p < 0.001 by one‐way ANOVA. *N* = 3 per group. CE, concentric exercise; EE, eccentric exercise; Sed, sedentary; TA, tibialis anterior.
**Figure S2:** EE promoted more EV secretion from skeletal muscle than CE during muscle repair. (a) The qPCR analysis of mRNA expression levels of Rab27 and Rab27b in the Ctrl, SED, CE and EE groups. Data are presented as mean ± SEM. *p < 0.05; **p < 0.01; ***p < 0.001 by one‐way ANOVA. N = 3 per group. CE, concentric exercise; EE, eccentric exercise; Sed, sedentary; TA, tibialis anterior.
**Figure S3:** Blockage of mEV secretion from skeletal muscle using GW4869 abolished the beneficial effects on muscle repair by eccentric exercise. (a) The qPCR analysis of mRNA expression levels of gene *Rab27a* and *Rab27b* in TA muscles treated with GW4869 or vehicle in the SED, CE and EE groups. (b) The qPCR analysis of mRNA expression levels of gene *Myog*, *Myod* and *eMyhc* in TA muscles treated with GW4869 or vehicle in the SED, CE and EE groups. (c) Western blot analysis and quantification of Myog was detected at the protein level with injecting GW4869 or vehicle in the SED, CE and EE groups. (d) The representative images of immunohistochemistry staining for Rab27a in TA muscle sections treated with GW4869 or vehicle in the SED, CE and EE groups and quantitative analysis of Rab27a‐positive staining among each group. Scale bar, 100 μm. Data are presented as mean ± SEM. *p < 0.05; **p < 0.01; ***p < 0.001 by two‐way ANOVA. *N* = 3 per group. CE, concentric exercise; EE, eccentric exercise; Sed, sedentary; TA, tibialis anterior.
**Figure S4:** The mEVs induced by eccentric exercise exerted more beneficial effects on promoting muscle repair than those generated by concentric exercise. Representative electron microscopy images of mEVs. Scale bar, 100 nm. The representative images of immunofluorescent staining for PKH26 (red) and Pax7 (green) in TA muscle sections of mice 24 h after intramuscular injection with PKH26‐labelled mEVs or PBS. Nuclei were labelled with DAPI (blue). Scale bar, 5 μm. (c) Western blot analysis and quantification of Myog. (d) The qPCR analysis for mRNA expression levels of genes including myog, myod and emyhc in TA muscles of the Ctrl, SED‐mEV, CE‐mEV and EE‐mEV group. (e) The representative curve for tetanic force generated during tetanic stimulation in EDL from the PBS, SED‐mEV, CE‐mEV and EE‐mEV groups. Data are presented as mean ± SEM. *p < 0.05; **p < 0.01; ***p < 0.001 by one‐way ANOVA. N = 3 per group. CE‐mEVs, mEVs derived from muscle of mice in the concentric exercise group; EE‐mEVs, mEVs derived from muscle of mice in the eccentric exercise group; SED‐mEVs, mEVs derived from muscle of mice in the resting group.
**Figure S5:** EE‐mEVs more effectively promoted myoblast differentiation than CE‐mEVs or SED‐mEVs. (a) The representative images of immunofluorescent staining for PKH26 (red) and phalloidin (green) in C2C12 cell 24 h after adding PKH26‐labelled mEVs or PBS. Nuclei were labelled with DAPI (blue). Scale bar, 5 μm. Data are presented as mean ± SEM. *p < 0.05; **p < 0.01; ***p < 0.001 by one‐way ANOVA. N = 3 per group.
**Figure S6:** Blockage of the lipid metabolism in muscle abolished the beneficial effects of CE/EE‐mEVs on muscle repair. (a) The western blotting analysis for the protein expression levels of Fasn in the Ctrl, BMS‐SED‐mEV, BMS‐CE‐mEV and BMS‐EE‐mEV groups. Data are presented as mean ± SEM. N = 3 per group. *p < 0.05; **p < 0.01; ***p < 0.001 by one‐way ANOVA.
**Figure S7:** Lipid metabolites enriched in mEVs contributed to muscle repair potentially through upregulating energy‐sensing genes associated with fatty acid metabolism in myoblasts. (a) Representative Western blot images showing the protein expression levels of p‐AMPK, AMPK and MyoD in the Ctrl, SED‐mEV, SED‐mEVs + PI, CE‐mEV, CE‐mEV + PI and EE‐mEV groups. Right panels show the quantitative analysis of the p‐AMPK/AMPK ratio and MyoD protein expression levels. (b) Left panel, representative images of immunofluorescence staining for Myod (red) and phalloidin (green) of the Ctrl, SED‐mEV, SED‐mEV + PI, CE‐mEV, CE‐mEV + PI and EE‐mEV groups. Nuclei were labelled with DAPI (blue). Scale bar, 50 μm. Right panel, quantitative analysis of Myod‐positive staining among each group. (c) Representative Western blot images showing the protein expression levels of p‐AMPK, AMPK and MyoD in the Ctrl, CC, EE‐mEV + CC and EE‐mEV groups. Right panels show the quantitative analysis of the p‐AMPK/AMPK ratio and MyoD protein expression levels. (d) Left panel, representative images of immunofluorescence staining for Myod (red) and phalloidin (green) of the Ctrl, CC, EE‐mEV + CC and EE‐mEV groups. Nuclei were labelled with DAPI (blue). Scale bar, 50 μm. Right panel, quantitative analysis of Myod‐positive staining among each group. Data are presented as mean ± SEM. N = 3 per group. *p < 0.05; **p < 0.01; ***p < 0.001 by one‐way ANOVA.
**Figure S8:** Representative images of JC‐1 staining. Red fluorescence represented intact mitochondria with normal membrane potential and green fluorescence represented impaired mitochondria with reduced membrane potential. Scale bar, 50 μm.
**Figure S9:** The schematic diagram depicting muscle‐derived extracellular vesicles in modulating satellite cell differentiation after injury. Both EE and CE exercise promote muscle repair after injury. Notably, EE induced a greater release of mEVs with exhibiting significant enrichment in lipid metabolites than CE, which can modulate satellite cells differentiation after injury.
**Table S1:** Aurora Scientific 1200A parameters.
**Table S2:** Key resources.
**Table S3:** Primer sequences.
**Table S4:** Exact p‐values.
**Table S5:** Top 10 most significantly altered lipid species.

## Data Availability

The original RNA‐seq data presented in this study have been deposited in the NCBI Gene Expression Omnibus (GEO) repository. The data are accessible under the accession number GSE326247 and will be made fully available to the public upon the publication of this article.

## References

[1] P. Edouard , P. Branco , and J. M. Alonso , “Muscle Injury Is the Principal Injury Type and Hamstring Muscle Injury Is the First Injury Diagnosis During Top‐Level International Athletics Championships Between 2007 and 2015,” British Journal of Sports Medicine 50 (2016): 619–630. 26887415 10.1136/bjsports-2015-095559

[2] N. Koulmann , H. Richard‐Bulteau , B. Crassous , et al., “Physical Exercise During Muscle Regeneration Improves Recovery of the Slow/Oxidative Phenotype,” Muscle & Nerve 55 (2017): 91–100. 27104889 10.1002/mus.25151

[3] M. S. Kang , J. Kim , and J. Lee , “Effect of Different Muscle Contraction Interventions Using an Isokinetic Dynamometer on Muscle Recovery Following Muscle Injury,” Journal of Exercise Rehabilitation 14 (2018): 1080–1084. 30656173 10.12965/jer.1836440.220 PMC6323341

[4] M. Roig , K. O'Brien , G. Kirk , et al., “The Effects of Eccentric Versus Concentric Resistance Training on Muscle Strength and Mass in Healthy Adults: A Systematic Review With Meta‐Analysis,” British Journal of Sports Medicine 43 (2009): 556–568. 18981046 10.1136/bjsm.2008.051417

[5] M. V. Franchi , N. D. Reeves , and M. V. Narici , “Skeletal Muscle Remodeling in Response to Eccentric vs. Concentric Loading: Morphological, Molecular, and Metabolic Adaptations,” Frontiers in Physiology 8 (2017): 447. 28725197 10.3389/fphys.2017.00447 PMC5495834

[6] P. Abreu and A. J. Kowaltowski , “Satellite Cell Self‐Renewal in Endurance Exercise Is Mediated by Inhibition of Mitochondrial Oxygen Consumption,” Journal of Cachexia, Sarcopenia and Muscle 11 (2020): 1661–1676. 32748470 10.1002/jcsm.12601 PMC7749620

[7] J. O. Brett , M. Arjona , M. Ikeda , et al., “Exercise Rejuvenates Quiescent Skeletal Muscle Stem Cells in Old Mice Through Restoration of Cyclin D1,” Nature Metabolism 2 (2020): 307–317. 10.1038/s42255-020-0190-0 PMC7323974 32601609

[8] X. Kang , J. Qian , Y. X. Shi , et al., “Exercise‐Induced Musclin Determines the Fate of Fibro‐Adipogenic Progenitors to Control Muscle Homeostasis,” Cell Stem Cell 31 (2024): 212–226 e7. 38232727 10.1016/j.stem.2023.12.011

[9] J. S. Kang , J. H. Kim , M. J. Kim , et al., “Exercise‐Induced CLCF1 Attenuates Age‐Related Muscle and Bone Decline in Mice,” Nature Communications 16 (2025): 4743. 10.1038/s41467-025-59959-w PMC12095553 40399268

[10] A. Safdar and M. A. Tarnopolsky , “Exosomes as Mediators of the Systemic Adaptations to Endurance Exercise,” Cold Spring Harbor Perspectives in Medicine 8, no. 3 (2018): a029827. 28490541 10.1101/cshperspect.a029827 PMC5830902

[11] A. Safdar , A. Saleem , and M. A. Tarnopolsky , “The Potential of Endurance Exercise‐Derived Exosomes to Treat Metabolic Diseases,” Nature Reviews. Endocrinology 12 (2016): 504–517. 10.1038/nrendo.2016.76 27230949

[12] X. Cao , L. Xue , X. Yu , et al., “Myogenic Exosome miR‐140‐5p Modulates Skeletal Muscle Regeneration and Injury Repair by Regulating Muscle Satellite Cells,” Aging (Albany NY) 16 (2024): 4609–4630. 38428405 10.18632/aging.205617 PMC10968704

[13] P. Ni , L. Yang , and F. Li , “Exercise‐Derived Skeletal Myogenic Exosomes as Mediators of Intercellular Crosstalk: A Major Player in Health, Disease, and Exercise,” Journal of Physiology and Biochemistry 79 (2023): 501–510. 37338658 10.1007/s13105-023-00969-x

[14] J. Luo , Q. Pu , and X. Wu , “Recent Advances of Exosomes Derived From Skeletal Muscle and Crosstalk With Other Tissues,” International Journal of Molecular Sciences 25, no. 20 (2024): 10877. 39456658 10.3390/ijms252010877 PMC11507631

[15] X. Shao , W. Gong , Q. Wang , et al., “Atrophic Skeletal Muscle Fibre‐Derived Small Extracellular Vesicle miR‐690 Inhibits Satellite Cell Differentiation During Ageing,” Journal of Cachexia, Sarcopenia and Muscle 13 (2022): 3163–3180. 36237168 10.1002/jcsm.13106 PMC9745557

[16] S. E. Alway , H. G. Paez , C. R. Pitzer , et al., “Mitochondria Transplant Therapy Improves Regeneration and Restoration of Injured Skeletal Muscle,” Journal of Cachexia, Sarcopenia and Muscle 14 (2023): 493–507. 36604839 10.1002/jcsm.13153 PMC9891964

[17] J. Touron , L. Maisonnave , J. P. Rigaudiere , et al., “Eccentric and Concentric Exercises Induce Different Adaptions in Adipose Tissue Biology,” Journal of Physiology and Biochemistry 79 (2023): 441–450. 36961725 10.1007/s13105-023-00956-2

[18] D. F. Martins , T. C. Martins , A. P. Batisti , et al., “Long‐Term Regular Eccentric Exercise Decreases Neuropathic Pain‐Like Behavior and Improves Motor Functional Recovery in an Axonotmesis Mouse Model: The Role of Insulin‐Like Growth Factor‐1,” Molecular Neurobiology 55 (2018): 6155–6168. 29250715 10.1007/s12035-017-0829-3

[19] Q. Zhang , D. K. Jeppesen , J. N. Higginbotham , J. L. Franklin , and R. J. Coffey , “Comprehensive Isolation of Extracellular Vesicles and Nanoparticles,” Nature Protocols 18 (2023): 1462–1487. 36914899 10.1038/s41596-023-00811-0 PMC10445291

[20] E. Serrano‐Pertierra , M. Oliveira‐Rodriguez , M. Matos , et al., “Extracellular Vesicles: Current Analytical Techniques for Detection and Quantification,” Biomolecules 10, no. 6 (2020): 824. 32481493 10.3390/biom10060824 PMC7357140

[21] C. Zhang , N. Cheng , B. Qiao , et al., “Age‐Related Decline of Interferon‐Gamma Responses in Macrophage Impairs Satellite Cell Proliferation and Regeneration,” Journal of Cachexia, Sarcopenia and Muscle 11 (2020): 1291–1305. 32725722 10.1002/jcsm.12584 PMC7567146

[22] Y. Liu , M. Ge , X. Xiao , et al., “Sarcosine Decreases in Sarcopenia and Enhances Muscle Regeneration and Adipose Thermogenesis by Activating Anti‐Inflammatory Macrophages,” Nature Aging 5 (2025): 1810–1827. 40550878 10.1038/s43587-025-00900-7

[23] D. M. Thomson , “The Role of AMPK in the Regulation of Skeletal Muscle Size, Hypertrophy, and Regeneration,” International Journal of Molecular Sciences 19, no. 10 (2018): 3125. 30314396 10.3390/ijms19103125 PMC6212977

[24] C. Chavarro‐Nieto , M. Beaven , N. Gill , and K. Hebert‐Losier , “Hamstrings Injury Incidence, Risk Factors, and Prevention in Rugby Union Players: A Systematic Review,” Physician and Sportsmedicine 51 (2023): 1–19. 34637371 10.1080/00913847.2021.1992601

[25] K. A. Murach , C. S. Fry , E. E. Dupont‐Versteegden , J. J. McCarthy , and C. A. Peterson , “Fusion and Beyond: Satellite Cell Contributions to Loading‐Induced Skeletal Muscle Adaptation,” FASEB Journal 35 (2021): e21893. 34480776 10.1096/fj.202101096R PMC9293230

[26] M. Becker , S. S. Joseph , F. Garcia‐Carrizo , et al., “Regulatory T Cells Require IL6 Receptor Alpha Signaling to Control Skeletal Muscle Function and Regeneration,” Cell Metabolism 35 (2023): 1736–1751. 37734370 10.1016/j.cmet.2023.08.010 PMC10563138

[27] K. M. Kim , G. D. Yoo , W. Heo , et al., “TAZ Stimulates Exercise‐Induced Muscle Satellite Cell Activation via Pard3‐p38 MAPK‐TAZ Signalling axis,” Journal of Cachexia, Sarcopenia and Muscle 14 (2023): 2733–2746. 37923703 10.1002/jcsm.13348 PMC10751443

[28] N. Srinivasan , K. Thangavelu , A. Sekar , B. Sanjeev , and S. Uthandi , “Aspergillus Caespitosus ASEF14, an Oleaginous Fungus as a Potential Candidate for Biodiesel Production Using Sago Processing Wastewater (SWW),” Microbial Cell Factories 20 (2021): 179. 34503534 10.1186/s12934-021-01667-3 PMC8427899

[29] D. C. Bittel and J. K. Jaiswal , “Contribution of Extracellular Vesicles in Rebuilding Injured Muscles,” Frontiers in Physiology 10 (2019): 828. 31379590 10.3389/fphys.2019.00828 PMC6658195

[30] S. Palermi , B. Massa , M. Vecchiato , et al., “Indirect Structural Muscle Injuries of Lower Limb: Rehabilitation and Therapeutic Exercise,” Journal of Functional Morphology and Kinesiology 6, no. 3 (2021): 75. 34564194 10.3390/jfmk6030075 PMC8482242

[31] L. Diao , Y. Peng , J. Wang , et al., “Eccentric Contraction Enhances Healing of the Bone‐Tendon Interface After Rotator Cuff Repair in Mice,” American Journal of Sports Medicine 51 (2023): 3835–3844. 37861235 10.1177/03635465231202901

[32] S. S. Rudisill , N. H. Varady , M. P. Kucharik , C. T. Eberlin , and S. D. Martin , “Evidence‐Based Hamstring Injury Prevention and Risk Factor Management: A Systematic Review and Meta‐Analysis of Randomized Controlled Trials,” American Journal of Sports Medicine 51 (2023): 1927–1942. 35384731 10.1177/03635465221083998

[33] G. Shefer , G. Rauner , P. Stuelsatz , D. Benayahu , and Z. Yablonka‐Reuveni , “Moderate‐Intensity Treadmill Running Promotes Expansion of the Satellite Cell Pool in Young and Old Mice,” FEBS Journal 280 (2013): 4063–4073. 23464362 10.1111/febs.12228 PMC3711960

[34] C. Zhao , Y. Wu , S. Zhu , H. Liu , and S. Xu , “Irisin Protects Musculoskeletal Homeostasis via a Mitochondrial Quality Control Mechanism,” International Journal of Molecular Sciences 25, no. 18 (2024): 10116. 39337601 10.3390/ijms251810116 PMC11431940

[35] H. A. Pahlavani , “Exercise Therapy to Prevent and Treat Alzheimer's Disease,” Frontiers in Aging Neuroscience 15 (2023): 1243869. 37600508 10.3389/fnagi.2023.1243869 PMC10436316

[36] C. Folgueira , L. Herrera‐Melle , J. A. Lopez , et al., “Remodeling p38 Signaling in Muscle Controls Locomotor Activity via IL‐15,” Science Advances 10 (2024): eadn5993. 39141732 10.1126/sciadv.adn5993 PMC11323882

[37] I. J. Vechetti, Jr. , T. Valentino , C. B. Mobley , and J. J. McCarthy , “The Role of Extracellular Vesicles in Skeletal Muscle and Systematic Adaptation to Exercise,” Journal of Physiology 599 (2021): 845–861. 31944292 10.1113/JP278929 PMC7363579

[38] L. Huang , M. Li , C. Deng , et al., “Potential Therapeutic Strategies for Skeletal Muscle Atrophy,” Antioxidants 12, no. 1 (2022): 44. 36670909 10.3390/antiox12010044 PMC9854691

[39] J. Luo , D. Zhang , Q. Pu , et al., “Skeletal Muscle‐Derived Exosomes Selectively Coated miRNAs and Participate in Myoblast Proliferation and Differentiation Mediated by miR‐4331‐3p,” International Journal of Biological Macromolecules 281 (2024): 136225. 39368577 10.1016/j.ijbiomac.2024.136225

[40] M. Qu , X. Zhou , X. Wang , and H. Li , “Lipid‐Induced S‐Palmitoylation as a Vital Regulator of Cell Signaling and Disease Development,” International Journal of Biological Sciences 17 (2021): 4223–4237. 34803494 10.7150/ijbs.64046 PMC8579454

[41] Y. Horibata , S. Mitsuhashi , H. Shimizu , et al., “The Phosphatidylcholine Transfer Protein StarD7 Is Important for Myogenic Differentiation in Mouse Myoblast C2C12 Cells and Human Primary Skeletal Myoblasts,” Scientific Reports 10 (2020): 2845. 32071354 10.1038/s41598-020-59444-y PMC7029042

[42] S. Tan‐Chen , J. Guitton , O. Bourron , H. Le Stunff , and E. Hajduch , “Sphingolipid Metabolism and Signaling in Skeletal Muscle: From Physiology to Physiopathology,” Front Endocrinol (Lausanne) 11 (2020): 491. 32849282 10.3389/fendo.2020.00491 PMC7426366

[43] Y. Kyriakidou , C. Wood , C. Ferrier , A. Dolci , and B. Elliott , “The Effect of Omega‐3 Polyunsaturated Fatty Acid Supplementation on Exercise‐Induced Muscle Damage,” Journal of the International Society of Sports Nutrition 18 (2021): 9. 33441158 10.1186/s12970-020-00405-1 PMC7807509

