# Supplementary material for: Skeletal Muscle–Derived Extracellular Vesicles During Eccentric and Concentric Exercise Promote Muscle Regeneration After Injury
Source: J Cachexia Sarcopenia Muscle. 2026 Aug 28;17(5):e70374. doi: 10.1002/jcsm.70374 (PMC13525339; doi:10.1002/jcsm.70374)
Supplement: Supplementary file 1 [file JCSM-17-e70374-s001.docx]

**Skeletal muscle-derived extracellular vesicles during eccentric and concentric exercise promote muscle regeneration after injury**

Yining Zhou^1,2,3^^,7^, Xiaoyan Shao^4,7^, Pan Zhang^1,2,3,7^, Jiaquan Lin^1,2,3^, Xiang Chen^1,2,3^, Xueying An^1,2,3^, Zhuoying Jiang^1,2,3^, Haosheng Wang^1,2,3^, Depeng Fang^1,2,3^, Yansi Xian^1,2,3^, Bin Liu^1,2,3^, Tao Shen^1,2,3^, Ying Chen^1,2,3^, Keran Li^1,2,3^, Hongpeng Liu^1,2,3^, Ying Li^5^, Qing Jiang^1,2,3*^, Baosheng Guo^1,2,3,6,8*^

^1^Division of Sports Medicine and Adult Reconstructive Surgery, Department of Orthopedic Surgery, Nanjing Drum Tower Hospital, Affiliated Hospital of Medical School, Nanjing University, 321 Zhongshan Road, Nanjing 210008, Jiangsu, PR China.

^2^State Key Laboratory of Pharmaceutical Biotechnology, Nanjing University, Nanjing, Jiangsu, PR China.

^3^Branch of National Clinical Research Center for Orthopedics, Sports Medicine and Rehabilitation, PR China.

^4^Department of Rheumatology and Immunology, Children's Hospital of Nanjing Medical University, Nanjing, China.

^5^Department of Orthopedics, Air Force Hospital of Eastern Theater Command, Nanjing University of Chinese Medicine, Nanjing, China.

^6^Department of Orthopedics, Nanjing Central Hospital, Nanjing, Jiangsu, China.

^7^These authors contributed equally

^8^Lead Contact

^*^Correspondence

Baosheng Guo: [[borisguo@nju.edu.cn]](mailto:[borisguo@nju.edu.cn])

Qing Jiang: [qingj@nju.edu.cn]

**Fig. S1 Eccentric exercise exerted more efficient effects than concentric exercise on promoting muscle repair following acute injury**

(a) The representative images of H&E staining of TA muscle in mice from the undamaged group (Ctrl), sedentary group (SED), concentric exercise group (CE), and eccentric exercise group (EE) in day3, day5, day7, day14. Scale bar, 100μm.

(b) sections of the Ctrl, SED, CE, and EE groups in day3, day5, day7, day14, with quantitative analysis of eMyhc-positive staining across groups. Nuclei were labeled with DAPI (blue). Scale bar, 50μm.

(c) Western blot analysis and quantification of Myod and Myog, which serve as indicators of new muscle fiber formation, were detected at the protein level in the SED, CE and EE groups.

(d)Left panel, the representative curve for tetanic force generated during tetanic stimulation in EDL from Ctrl, SED, CE and EE groups in Day7. Right panel, the representative curve for tetanic force generated during tetanic stimulation in EDL from Ctrl, SED, CE and EE groups in Day14.

Notes: Data are presented as means ± SEM. *P < 0.05, **P < 0.01, ***P < 0.001 by one-way ANOVA. N = 3 per group. Sed, sedentary; CE, concentric exercise; EE, eccentric exercise; TA, tibialis anterior.

**Fig.S2 EE promoted more EVs secretion from skeletal muscle than CE during muscle repair**

(a) The qPCR analysis of mRNA expression levels of *Rab27* and *Rab27b* in Ctrl, SED, CE, and EE groups.

Notes: Data are presented as means ± SEM. *P < 0.05, **P < 0.01, ***P < 0.001 by one-way ANOVA. N = 3 per group. Sed, sedentary; CE, concentric exercise; EE, eccentric exercise; TA, tibialis anterior.

**Fig.S3 Blockage of mEVs secretion from skeletal muscle using GW4869 abolished the beneficial effects on muscle repair by eccentric exercise**

(a) The qPCR analysis of mRNA expression levels of gene *Rab27a* and *Rab27b* in TA muscles treated with GW4869 or vehicle in the SED, CE, and EE groups.

(b) The qPCR analysis of mRNA expression levels of gene *Myog, Myod* and *eMyhc* in TA muscles treated with GW4869 or vehicle in the SED, CE, and EE groups.

(c) Western blot analysis and quantification of Myog was detected at the protein level with injecting GW4869 or vehicle in the SED, CE, and EE groups.

(d) The representative images of immunohistochemistry staining for Rab27a in TA muscle sections treated with GW4869 or vehicle in the SED, CE, and EE groups and quantitative analysis of Rab27a-positive staining among each group. Scale bar, 100μm.

Notes: Data are presented as means ± SEM. *P < 0.05, **P < 0.01, ***P < 0.001 by two-way ANOVA. N = 3 per group. Sed, sedentary; CE, concentric exercise; EE, eccentric exercise; TA, tibialis anterior.

**Fig.S4 The mEVs induced by eccentric exercise exerted more beneficial effects on promoting muscle repair than those generated by concentric exercise**

(a) Representative electron microscopy images of mEVs. scale bar, 100 nm.
(b) The representative images of immunofluorescent staining for PKH26 (red) and Pax7 (green) in TA muscle sections of mice 24 h after intramuscular injection with PKH26-labelled mEVs or PBS. Nuclei were labeled with DAPI (blue). Scale bar, 5μm.

(c) Western blot analysis and quantification of Myog.

(d) The qPCR analysis for mRNA expression levels of genes including *myog*, *myod* and *emyhc* in TA muscles of the Ctrl, SED-mEVs, CE-mEVs and EE-mEVs group.

(e) the representative curve for tetanic force generated during tetanic stimulation in EDL from PBS, SED-mEVs, CE-mEVs and EE-mEVs groups.

Notes: Data are presented as means ± SEM. *P < 0.05, **P < 0.01, ***P < 0.001 by one-way ANOVA. N = 3 per group. SED-mEVs, mEVs derived from muscle of mice in the resting group; CE-mEVs, mEVs derived from muscle of mice in the concentric exercise group; EE-mEVs, mEVs derived from muscle of mice in the eccentric exercise group.

**Fig.S5 EE-mEVs more effectively promoted myoblast differentiation than CE-mEVs or SED-mEVs**

(a) The representative images of immunofluorescent staining for PKH26 (red) and phalloidin (green) in C2C12 cell 24 h after adding PKH26-labelled mEVs or PBS. Nuclei were labeled with DAPI (blue). Scale bar, 5μm.

Notes: Data are presented as means ± SEM. *P < 0.05, **P < 0.01, ***P < 0.001 by one-way ANOVA. N = 3 per group.

**Fig.S6 Blockage of the lipid metabolism in muscle abolished the beneficial effects of CE/EE-mEVs on muscle repair**

(a) The western blotting analysis for the protein expression levels of Fasn in the Ctrl, BMS-SED-mEVs, BMS-CE-mEVs and BMS-EE-mEVs groups.

Notes: Data are presented as means ± SEM. N = 3 per group. *P < 0.05, **P < 0.01, ***P < 0.001 by one-way ANOVA.

**Fig.S7 Lipid metabolites enriched in mEVs contributed to muscle repair potentially through upregulating energy-sensing genes associated with fatty acid metabolism in myoblasts.**

(a) Representative Western blot images showing the protein expression levels of p-AMPK, AMPK, and MyoD in Ctrl, SED-mEVs, SED-mEVs+PI, CE-mEVs, CE-mEVs+PI, and EE-mEVs groups. Right panels show the quantitative analysis of the p-AMPK/AMPK ratio and MyoD protein expression levels.

(b)	Left panel, representative images of immunofluorescence staining for Myod (red) and phalloidin (green) of the Ctrl, SED-mEVs, SED-mEVs+PI, CE-mEVs, CE-mEVs+PI, and EE-mEVs groups. Nuclei were labeled with DAPI (blue). Scale bar, 50μm. Right panel, quantitative analysis of Myod-positive staining among each group.

(c) Representative Western blot images showing the protein expression levels of p-AMPK, AMPK, and MyoD in Ctrl, CC, EE-mEVs+CC and EE-mEVs groups. Right panels show the quantitative analysis of the p-AMPK/AMPK ratio and MyoD protein expression levels.

(d)	Left panel, representative images of immunofluorescence staining for Myod (red) and phalloidin (green) of the Ctrl, CC, EE-mEVs+CC and EE-mEVs groups. Nuclei were labeled with DAPI (blue). Scale bar, 50μm. Right panel, quantitative analysis of Myod-positive staining among each group.

Notes: Data are presented as means ± SEM. N = 3 per group. *P < 0.05, **P < 0.01, ***P < 0.001 by one-way ANOVA.

**Fig.S8 Representative images of JC-1 staining.** Red fluorescence represented intact mitochondria with normal membrane potential and green fluorescence represented impaired mitochondria with reduced membrane potential. Scale bar, 50 μm.

**Fig.S9 The schematic diagram depicting** **muscle-derived extracellular vesicles in modulating satellite cells differentiation after injury.** Both EE and CE exercise promote muscle repair after injury. Notably, EE induced a greater release of mEVs with exhibiting significant enrichment in lipid metabolites than CE, which can modulate satellite cells differentiation after injury.

**Table S1. Aurora Scientific 1200A Parameters**

|  | Stimulation frequency | Plateau  duration | Stretch displacement | Stretch ramp duration |
| --- | --- | --- | --- | --- |
| EE | 100Hz | 500ms | 10% | 50ms |
| CE | 100Hz | 500ms | -10% | 50ms |

**Table S2. Key Resources**

| **Reagent or Resource** | **Source** | **Identifier** |
| --- | --- | --- |
| COL1A1 Rabbit mAb  HRP-conjugated Goat anti-Rabbit IgG (H+L)  Mouse anti-emyhc  Rabbit anti-Laminin  Goat anti-Mouse Secondary Antibody, Alexa Fluor™ 488  Goat anti-Rabbit Secondary Antibody, Alexa Fluor™ 488  Donkey anti-Rabbit Secondary Antibody, Alexa Fluor™ 594  Goat anti-Mouse Secondary Antibody, Alexa Fluor™ 594  α-Tubulin Rabbit pAb  Anti-Myogenin antibody  MYOD1 Rabbit pAb  Anti-CD63 antibody  Anti-CD9 antibody  ALIX Rabbit mAb  TSG101 Rabbit mAb  RAB27A Rabbit mAb | ABclonal  ABclonal  DSHB  ABclonal  Invitrogen  Invitrogen  Invitrogen  Invitrogen  ABclonal  ABCAM  proteintech  ABCAM  ABCAM  ABclonal  proteintech  Abclonal | A26946  AS014  F1.652  A5968  A-11001  A-11008  A-21207  A-11005  AC007  ab 124800  18943-1-AP  ab315108  ab223052  A25326  28283-1-AP  A23993 |
| Dichlorobarium  Antifade Mounting Medium with DAPI  QuickBlock Blocking Buffer for Immunol Staining  Y-Tec Ready-to-use Blocking Buffer  ChamQ SYBR Color qPCR Master Mix  HisyGo RT Red SuperMix for qPCR  PBS  Krebs-Ringer Buffer  GW4869 | Sigma  Beyotime  Beyotime  Yoche  Vazyme  Vazyme  Servicebio  Solarbio  Selleck | 342920-10G  P0131-25ml  P0260  YWB0501  Q411-02  RT101-01  G4202-500ml  G0430  Catalog No. S7609 |

**Table S3. Primer sequences**

| **Name** | **Primer sequences** |
| --- | --- |
| Rab27a | Forward: 5’- TCGGATGGAGATTACGATTACCT-3’ |
|  | Reverse: 5’- TTTTCCCTGAAATCAATGCCCA-3’ |
| Rab27b | Forward: 5’- CGTCAGGAAAAGCGTTTAAGGT-3’ |
|  | Reverse: 5’- AGAAGCTCTGTTGACTGGTGA-3’ |
| MyoD1 | Forward: 5’- GACGGCTCTCTCTGCTCC-3’ |
|  | Reverse: 5’- AAGTGTGCGTGCTCCTCC-3’ |
| MyoG | Forward: 5’- CAGTGAATGCAACTCCCACAG-3’ |
|  | Reverse: 5’- TGGACGTAAGGGAGTGCAGA-3’ |
| MyHC | Forward: 5’- GAGTTCATTGACTTCGGGATGG-3’ |
|  | Reverse: 5’- TGCTGCTCATACAGCTTGTTCTTG-3’ |
| AP2 | Forward: 5’- AAGGTGAAGAGCATCATAACCC-3 |
|  | Reverse: 5’- TCACGCCTTTCATAACACATTC-3’ |
| FAS | Forward: 5’- TGGAGGTGGTGATAGCCGGTAT-3  Reverse: 5’- CTGGGTAATCCATAGAGCCCAG-3 |
| LPL | Forward: 5’- GGGAGTTTGGCTCCAGAGTTT-3  Reverse: 5’- TGTGTCTTCAGGGGTCCTTAG-3 |
| FAT | Forward: 5’-ATGGGCTGTGATCGGAACTG-3  Reverse: 5’-GTCTTCCCAATAAGCATGTCTC-3 |
| PPAR α | Forward: 5’-AGAGCCCCATCTGTCCTCTC-3  Reverse: 5’-ACTGGTAGTCTGCAAAACCAAA-3 |
| PPAR β | Forward: 5’-TCCATCGTCAACAAAGACGGG-3  Reverse: 5’-ACTTGGGCTCAATGATGTCAC-3 |
| PPAR γ | Forward: 5’-TCGCTGATGCACTGCCTATG-3  Reverse: 5’-GAGAGGTCCACAGAGCTGATT-3 |
| Gapdh | Forward: 5’-AGGTCGGTGTGAACGGATTTG-3’ |
|  | Reverse: 5’-TGTAGACCATGTAGTTGAGGTCA-3’ |

**Supplementary Table (Table S4). Exact p-values**

| **Figure Panel** | **Gene/Protein** | **Comparison and P-value** |
| --- | --- | --- |
| Figure1c | Collagen I | SED vs CE, P <0.001; SED vs EE, P <0.001; CE vs EE, P =0.0014 |
| Figure1e | emyhc | SED vs CE, P = 0.1198; SED vs EE, P = 0.0161; CE vs EE, P < 0.001 |
| Figure1e | Myog | SED vs CE, P =0.0523; SED vs EE, P = 0.0001; CE vs EE, P = 0.0303 |
| Figure1e | Myod | SED vs CE, P = 0.0305; SED vs EE, P = 0.001; CE vs EE, P = 0.1089 |
| Figure1f | eMyhc | SED vs CE, P =0.0001; SED vs EE, P <0.001; CE vs EE, P = 0.0013 |
| Figure2a | Rab27a | SED vs CE, P =0.0717; SED vs EE, P =0.0363; CE vs EE, P =0.8488 |
| Figure2f | EDL | SED vs CE, P =0.0023; SED vs EE, P <0.001; CE vs EE, P =0.0374 |
| Figure2f | SOL | SED vs CE, P =0.8139; SED vs EE, P =0.0290; CE vs EE, P=0.0346 |
| Figure2f | TA | SED vs CE, P <0.001; SED vs EE, P <0.001; CE vs EE, P <0.001 |
| Figure3b | CD63 | SED+Vehicle vs SED+GW4869, P <0.001;  CE+Vehicle vs CE+GW4869, P<0.001;  EE+Vehicle vs EE+GW4869, P <0.001 |
| Figure3d | Collagen I | SED+Vehicle vs SED+GW4869, P =0.9495;  CE+Vehicle vs CE+GW4869, P <0.001;  EE+Vehicle vs EE+GW4869, P <0.001 |
| Figure3f | eMyhc | SED+Vehicle vs SED+GW4869, P <0.001;  CE+Vehicle vs CE+GW4869, P <0.001;  EE+Vehicle vs EE+GW4869, P <0.001 |
| Figure4d | Collagen I | SED-mEVs vs CE-mEVs, P =0.0013; SED-mEVs vs EE-mEVs, P<0.001; CE-mEVs vs EE-mEVs, P=0.0200 |
| Figure4f | eMyhc | SED-mEVs vs CE-mEVs, P =0.0193; SED-mEVs vs EE-mEVs, P =0.0012; CE-mEVs vs EE-mEVs, P =0.0593 |
| Figure6a | Myod | SED-mEVs vs CE-mEVs, P =0.0440; SED-mEVs vs EE-mEVs, P =0.0005; CE-mEVs vs EE-mEVs, P =0.0065 |
| Figure6b | Myog | SED-mEVs vs CE-mEVs, P =0.0050; SED-mEVs vs EE-mEVs, P< 0.001; CE-mEVs vs EE-mEVs, P= 0.0018 |
| Figure6c | Myod | SED-mEVs vs CE-mEVs, P =0.3315; SED-mEVs vs EE-mEVs, P< 0.001; CE-mEVs vs EE-mEVs, P< 0.001 |
| Figure6d | Myod | SED-mEVs vs CE-mEVs, P = 0.7217; SED-mEVs vs EE-mEVs, P = 0.0994; Ctrl vs EE-mEVs, P =0.0151 |
| Figure7d | Myod | BMS-SED-mEVs vs BMS-CE-mEVs, P = 0.1608  BMS-SED-mEVs vs BMS-EE-mEVs, P = 0.2625  BMS-CE-mEVs vs BMS-EE-mEVs,, P = 0.9821 |
| Figure8e | ATP | Ctrl vs SED-mEVs, P = 0.5187; SED-mEVs vs EE-mEVs, P = 0.0425; Ctrl vs EE-mEVs, P = 0.0057 |

**Table S5.** Top 10 most significantly altered lipid species

| **Rank** | **Lipid Species** | **Lipid Class** | **SED MEAN** | **CE MEAN** | **EE MEAN** | **P-value** |
| --- | --- | --- | --- | --- | --- | --- |
| 1 | PC(37:2) | Phosphatidylcholine (PC) | 1.1137E-05 | 1.4578E-05 | 1.7511E-05 | 0.00154 |
| 2 | PC(31:0) | Phosphatidylcholine (PC) | 1.9199E-04 | 1.9693E-04 | 2.5155E-04 | 0.00156 |
| 3 | SM(d18:1/18:0) | Sphingomyelin (SM) | 2.7105E-04 | 1.9351E-04 | 3.6853E-04 | 0.00687 |
| 4 | SM(d18:1/18:0) | Sphingomyelin (SM) | 3.0969E-05 | 2.1768E-05 | 4.1544E-05 | 0.00799 |
| 5 | PC(18:1(6Z)/18:1(6Z)) | Phosphatidylcholine (PC) | 1.6718E-03 | 1.3748E-03 | 1.6631E-03 | 0.00941 |
| 6 | 1-Hexadecyl-2-(9Z-octadecenoyl)-sn-glycero-3-phosphocholine | Phosphatidylcholine (PC) | 8.5128E-05 | 6.1729E-05 | 7.2133E-05 | 0.00985 |
| 7 | PC(18:0/18:3(6Z,9Z,12Z)) | Phosphatidylcholine (PC) | 2.58E-04 | 2.89E-04 | 3.26E-04 | 0.01267 |
| 8 | SM(d18:1/17:0) | Sphingomyelin (SM) | 6.6893E-06 | 7.7965E-06 | 1.2820E-05 | 0.02618 |
| 9 | 1,2-Dipalmitoyl-sn-glycero-3-phosphoethanolamine | Phosphatidylethanolamine (PE) | 2.1108E-05 | 2.1384E-05 | 2.8304E-05 | 0.02796 |
| 10 | PI(18:1/18:1) | Phosphatidylinositol (PI) | 1.5758E-06 | 6.5779E-06 | 7.0762E-06 | 0.02991 |
